# Gestational Low Protein Diet Modulation on miRNA Transcriptome and Its Target During Fetal and Breastfeeding Nephrogenesis

**DOI:** 10.3389/fphys.2021.648056

**Published:** 2021-06-22

**Authors:** Letícia de Barros Sene, Gabriela Leme Lamana, Andre Schwambach Vieira, Wellerson Rodrigo Scarano, José Antônio Rocha Gontijo, Patrícia Aline Boer

**Affiliations:** ^1^Department of Structural and Functional Biology, Institute of Biosciences, São Paulo State University (UNESP), Botucatu, Brazil; ^2^Fetal Programming and Hydroelectrolyte Metabolism Laboratory, Nucleus of Medicine and Experimental Surgery, Department of Internal Medicine, FCM, Campinas, Brazil; ^3^Department of Structural and Functional Biology, Biology Institute, State University of Campinas (UNICAMP), Campinas, Brazil

**Keywords:** fetal programming, gestational low-protein intake, nephrogenesis, metanephric mesenchyme, low nephron number, miRNA

## Abstract

**Background:**

The kidney ontogenesis is the most structurally affected by gestational protein restriction, reducing 28% of their functional units. The reduced nephron number is predictive of hypertension and cardiovascular dysfunctions that are generally observed in the adult age of most fetal programming models. We demonstrate miRNAs and predict molecular pathway changes associated with reduced reciprocal interaction between metanephros cap (CM) and ureter bud (UB) and a 28% decreased nephron stem cells in the 17 gestational days (17GD) low protein (LP) intake male fetal kidney. Here, we evaluated the same miRNAs and predicted targets in the kidneys of 21GD and at 7 days of life (7DL) LP offspring to elucidate the molecular modulations during nephrogenesis.

**Methods:**

Pregnant Wistar rats were allocated into two groups: NP (regular protein diet- 17%) or LP (diet-6%). miRNA transcriptome sequencing (miRNA-Seq) was performed on the MiSeq platform from 21GD and 7DL male offspring kidneys using previously described methods. Among the top 10 dysfunctional regulated miRNAs, we validated 7 related to proliferation, differentiation, and apoptosis processes and investigated predicted target genes and proteins by RT-qPCR and immunohistochemistry.

**Results:**

In 21GD, LP fetuses were identified alongside 21 differently expressed miRNAs, of which 12 were upregulated and 9 downregulated compared to age-matched NP offspring. In 7-DL LP offspring, the differentially expressed miRNAs were counted to be 74, of which 46 were upregulated and 28 downregulated. The curve from 17-GD to 7-DL shows that mTOR was fundamental in reducing the number of nephrons in fetal kidneys where the mothers were subjected to a protein restriction. IGF1 and TGFβ curves also seemed to present the same mTOR pattern and were modulated by miRNAs 181a-5p, 181a-3p, and 199a-5p. The miRNA 181c-3p modulated SIX2 and Notch1 reduction in 7-DL but not in terms of the enhanced expression of both in the 21-GD, suggesting the participation of an additional regulator. We found enhanced Bax in 21-GD; it was regulated by miRNA 298-5p, and Bcl2 and Caspase-3 were controlled by miRNA (by 7a-5p and not by the predicted 181a-5p). The miRNA 144-3p regulated BCL6, which was enhanced, as well as Zeb 1 and 2 induced by BCL6. These results revealed that in 21GD, the compensatory mechanisms in LP kidneys led to the activation of UB ramification. Besides, an increase of 32% in the CM stem cells and a possible cell cycle halt of renal progenitor cells, which remaining undifferentiated, were observed. In the 7DL, much more altered miRNA expression was found in LP kidneys, and this was probably due to an increased maternal diet content. Additionally, we verified the activation of pathways related to differentiation and consumption of progenitor cells.

## Introduction

Maternal nutritional restriction results in several critical changes in the fetal organ and system during the development stages, which may cause irreversible disorders in adult life (Langley-Evans, 2006). Fetal programming is characterized as any psychological and nutritional stress during development, which leads to long-term effects on kidney structure and function disorders with an increasing predictive chance to develop chronic renal disease (Lucas, 1998). Disturbance in fetal programming results in low birth weight, fewer nephrons, and increased risk of cardiovascular and renal disorders in adulthood (Mesquita et al., 2010a, b; Sene et al., 2013, 2018, 2021). The low nephron number is related to hypertension, and in hypertensive patients, approximately 46% of the number of nephrons is reduced (Mackenzie et al., 1996). Prior experimental studies from our lab and other authors have demonstrated lower birth weight, 28% fewer nephrons, reduced renal salt excretion, chronic renal failure, and enhanced systolic pressure from 8 to 16 weeks of life in gestational low-protein (LP) intake compared to standard (NP) protein intake offspring in adulthood (Schreuder et al., 2006; Mesquita et al., 2010a, b; Sene et al., 2013, 2018, 2021). However, information regarding the molecular mechanisms of the etiopathogenesis of nephrogenesis cessation is still scarce. Recently, Huang et al. showed evidence indicating epigenetic mechanisms controlling the nephrogenesis process.

Nephrogenesis involves fine control of gene expression, protein synthesis, tissue remodeling, and cell fates of the different kidney progenitor cells (Huang et al., 2020). During renal ontogenesis, nephron stem cell renewal and differentiation are too controlled to generate an adequate number of nephrons. These kidney nephron numbers are defined by a closed interaction among ureter bud (UB) and metanephros mesenchyme (MM) progenitor cells (Grobstein, 1955; Saxen and Sariola, 1987; Pan et al., 2017). Signals from MM induce UB-stimulated growth and branching of the tubule system. In turn, MM proliferation and differentiation, constituting a mesenchymal cap (CM), is mediated by UB ends (Phua et al., 2015). There has been serious interest in the role of epigenetic changes, concerning the long-term effects of prenatal stress, on fetal development (Monk et al., 2012). MicroRNAs (miRNAs) are genome-encoded small non-coding RNAs of approximately 22 nucleotides in length, and they play an essential role in the post-transcriptional regulation of target gene expression (Ambros, 2004; Bartel, 2004; Nilsen, 2007). Studies indicate that miRNAs are involved in many regulatory biological networks during development and cell physiology. Deregulation in their expression has been observed in several pathologies (Bushati and Cohen, 2007; Chang and Mendell, 2007). Thus, miRNA characterization is indispensable for nephron development and may help us understand gene regulation and cellular proliferation, differentiation, and apoptosis and explain pathophysiology, including kidney disorders (Chu and Rana, 2007; Harvey et al., 2008; Kim et al., 2009; Li et al., 2010; Chu et al., 2014). However, reduced miRNA expression in MM progenitor cells may decrease cell proliferation, resulting in early differentiation and a reduced number of nephrons (Ho et al., 2011; Nagalakshmi et al., 2011). This phenomenon is characterized by increased apoptosis and high Bim expression in progenitor cells. Thus, miRNAs modulate the balance between apoptosis and proliferation of these metanephric primary cells (Ho et al., 2011). We recently demonstrated in the fetus at 17 days of gestation (GD) protein-restricted male fetus changes in metanephros miRNAs and predicted mRNA expression that encodes proteins related to nephrogenesis a 28% reduction in nephrogenic stem cells in the cap metanephric (CM). We suggested that miRNAs, mRNAs, and protein disruption could have reduced proliferation and promote early cell differentiation (Sene et al., 2021). The rats are remaining the nephrogenesis after birth. Brown et al. (2016) showed in Sprague-Dawley rats that renal papilla is mature on day 7 of life (7DL), but cortex and medulla maturation occur, respectively, on 21 and 30DL. Additionally, Schreuder et al. (2006) demonstrated a 25% reduced nephron number over the breastfeeding period in offspring submitted to nutritional stress. Thus, in the present study, we evaluated during the last gestational day and during breastfeeding the miRNAs transcriptome and predicted targets of those more altered on the kidney of male offspring submitted to gestational low protein diet to elucidate the time-course of molecular modulations during nephrogenesis.

## Materials and Methods

### Animal and Diets

The experiments were conducted on age-matched female and male rats of sibling-mated Wistar *HanUnib*rats (250–300 g) that were allowed free access to water and standard rodent chow (Nuvital, Curitiba, PR, Brazil). The Institutional Ethics Committee (#446-CEEA/UNESP) approved the experimental protocol, and the general guidelines established by the Brazilian College of Animal Experimentation were followed throughout the investigation. At 12 weeks of age, the animals were mated, and the day that sperm were seen in the vaginal smear was designated as day 1 of pregnancy. Then, dams were maintained *ad libitum* throughout the entire pregnancy on an isocaloric rodent laboratory chow with either standard protein content [NP, *n* = 20] (17% protein) or low protein content [LP, *n* = 20] (6% protein) diets. The NP and LP maternal food consumption were determined daily (subsequently normalized for body weight), and the body weight of dams was recorded weekly in both groups. At 21 days of gestation (GD), in the dams on anesthesia (75 mg/kg ketamine and 10 mg/kg xylazine), the fetuses were removed and sacrificed. The fetuses were weighed, and the kidneys were collected, weighed, and processed for Next Generation Sequencing (NGS), RT-qPCR, and immunohistochemistry analyses. An additional group of dams was maintained on the NP and LP diets throughout the entire pregnancy. After delivery, the male pups were weighed at the birth, and NP and LP dams returned to standard protein content chow intake. At 21 GD, the kidneys from male NP and LP offspring were removed, weighed, and processed for NGS, RT-qPCR, and morphological analyses.

### Sexing Determination

The present study was performed only in male 17-GD offspring, and the sexing was determined by Sry conventional PCR (Polymerase Chain Reaction) sequence analysis. The DNA was extracted by enzymatic lysis with proteinase K and Phenol-Chloroform. For reaction, the Master Mix Colorless—Promega was used, with the manufacturer’s cycling conditions. The Integrated DNA Technologies (IDT) synthesized the primer following sequences below:

1. Forward: 5′-TACAGCCTGAGGACATATTA-3′

2. Reverse: 5′-GCACTTTAACCCTTCGATTAG-3′.

### Total RNA Extraction

RNA was extracted from NP (21GD, *n* = 4 and 7DL *n* = 4, from different mothers) and LP (21GD, *n* = 4 and 7DL *n* = 4, from different mothers) whole kidneys using Trizol reagent (Invitrogen), according to the instructions specified by the manufacturer. Total RNA quantity was determined by the absorbance at 260 nm using a nanoVue spectrophotometer (GE Healthcare, United States). RNA Integrity was ensured by obtaining an RNA Integrity Number - RIN > 8 with Agilent 2100 Bioanalyzer (Agilent Technologies, Germany).

#### miRNA-Seq and Data Analysis

Sequencing was performed on the MiSeq platform (Illumina). The protocol followed the manufacturer’s instructions available in http://www.illumina.com/documents//products/datasheets/datasheet_truseq_sample_prep_kits.pdf. Briefly, the sequencing includes library construction, and this used 1μg total RNA. In this step, the adapters are connected (3′ and 5′). After ligation of adapters, a reverse transcription reaction was performed to create cDNA. It was then amplified by a standard PCR reaction, which uses primers containing a sequence index for sample identification—this cDNA library, subjected to agarose gel electrophoresis for miRNA isolation. After quantitation, the library concentration was normalized to 2 nM using 10 nM Tris-HCl, pH 8.5, and transcriptome sequencing was performed by MiSeq Reagent Kit v2 (50 cycles). The library construction used the TruSeq Small RNA Library Preparation Kit (catalog number RS-200-0012). Approximately 530,000 reads/samples (21DG and 7DPN) were obtained with a size of 1.35GB. The study has used Genome Reference version rn5 from the UCSC Genome Browser or (UCSC rn5) for *Rattus norvegicus*. The miRNA sequences were acquired from miRBase v21. The alignment was done by Bowtie software (version 0.12.8), and the Differential Expression was obtained by way of the Small RNA software (BaseSpace Workflow) version 1.0.0.0. and the list of all sequenced miRNAs defined by DESeq2 (version 1.0.17). Also, the sequencing data were deposited in the Bioproject repository https://www.ncbi.nlm.nih.gov/bioproject/PRJNA694197 (NCBI) with the access number: PRJNA694197. Data analysis was performed in collaboration with Tao Chen, Ph.D., from the Division of Genetic and Molecular Toxicological, National Center for Toxicological Research, Jefferson, AR, United States. The data from the Next Generation Sequencing (NGS) of miRNAs were generated in a FASTAQ format and imported into BaseSpace.com (Illumina, United States). The data quality was evaluated using the base calling CASAVA software developed by the manufacturer (Illumina). The analyses were carried out using a BaseSpace miRNA Analysis (from the University of Toronto, Canada) and the sequence mapping of different miRNAs carried out by Small RNA (Illumina, United States) for the rat genome. The differentially expressed miRNA study was analyzed using Ingenuity Pathway Analysis software (Ingenuity, United States).

### miRNA Expression Validation

Among the most differentially expressed miRNA, we selected seven (miR-127-3p, -144-3p, -298-5p, let-7a-5p, Lin28b, -181a-5p, -181c-3p, and -199a-5p) for expression analysis. Briefly, 450 ng RNA was reverse transcribed, without pre-amplification, using a TaqMan^®^ MicroRNA Reverse Transcription Kit according to the manufacturer’s guidelines. Complementary DNA (cDNA) was amplified using TaqMan MicroRNA Assays (Life Technologies, United States) with *TaqMan^®^ Universal PCR Master Mix, No AmpErase^®^ UNG* (2x) on StepOnePlusTM Real-Time PCR System (Applied BiosystemsTM) according to the manufacturer’s instructions. Data analysis was performed using relative gene expression evaluated using the comparative quantification method (Pfaffl, 2001). The U6 and U87 gene was used as a reference gene. All relative quantifications were evaluated using the DataAssist software, v 3.0, using the ΔΔCT method. miRNA data have been generated following the MIQE guidelines (Bustin et al., 2009).

### RT-qPCR of Predicted Target and ZEB1/2 Genes

For the cDNA synthesis, the High Capacity cDNA reverse transcription kit (Life Technologies, United States) was used. To analyze the level of expression of 22 genes (Bax, Bim, Caspase-3, Collagen 1, GDNF, PCNA, TGFβ-1, Bcl-2, Bcl-6, c-myc, c-ret, cyclin A, Map2k2, PRDM1, Six-2, Ki67, MTOR, β-catenin, ZEB1, ZEB2, NOTCH1, and IGF1), the reaction of RT-qPCR was performed with SYBR Green Master Mix (Life Technologies, United States), using primers specific for each gene, provided by IDT^®^ Integrated DNA Technologies (Table 1). The reactions were done in a total volume of 20 μL using 2 μL of cDNA (diluted 1:30), 10 μL SYBER Green Master Mix (Life Technologies, United States), and 4 μL of each specific primer (5 nM). Amplification and detection were performed using the StepOnePlusTM Real-Time PCR System (Applied BiosystemsTM). Ct values were converted to relative expression values using the ΔΔCt method with offspring kidneys data normalized to GAPDH as a reference gene.

**TABLE 1:** Dilution of antibodies used in immunohistochemistry.

Antibody	Dilution	Company
Anti-Six2 (11562-1-AP)	1:50	Proteintech
Anti-c-myc (NBP1-19671)	1:150	Novus Biologicals
Anti-Ki67 (ab16667)	1:100	Abcam
Anti-Bcl2 (ab7973)	1:100	Abcam
Anti-TGFβ-1 (sc-146)	1:50	Santa Cruz
Anti-B -catenina (ab32572)	1:500	Abcam
Anti-Zeb1 (sc-10572)	1:50	Santa Cruz
Anti-Zeb2 (sc-48789)	1:50	Santa Cruz
Anti-VEGF (NB100-664)	1:50	Novus Biologicals
Anti-Caspase-3 clivada (9664)	1:200	Cell Signaling
Anti-Ciclina A (sc-31085)	1:50	Santa Cruz
Anti-WT1 (sc-192)	1:50	Santa Cruz
Anti-mTOR	1:100	Abcam

### Area and Cells Quantification

Hematoxylin-eosin stained paraffin sections (5NP and 5LP from different mothers) were used to measure renal, cortical, and medullar areas in the kidneys from 21GD and 7DL animals. The CAP areas and cell analysis were performed by microscopic fields digitized (Olympus BX51) using CellSens Dimension or *ImageJ* software evaluating images of the histological section in HE. The relative percentages of the cortical and medullary area relative to the total renal area, previously determined, were also determined. The whole kidney area corresponds to the determination of measures of the entire cut, that is, the sum of cortical and medullary area. Therefore, we reiterate that the histological section’s whole CAP regions were evaluated for each animal (*n* = 4) in both groups. For this procedure, the kidneys were sectioned longitudinally in half and embedded in paraffin each half. Then, the microtome cuts were made after the block was trimmed and stained in HE. The studies were carried out blindly and similarly for both groups of animals (NP and LP).

### Immunohistochemistry

After euthanasia, the kidneys from NP (21GD, *n* = 5 and 7DL *n* = 5, from different mothers) and LP (21GD, *n* = 5 and 7DL *n* = 5, from different mothers) were removed and fixed (4% paraformaldehyde in 0.1 M phosphate buffer pH 7.4) for 4 h. After, they were washed in running water and followed by 70% alcohol until processed. The materials were dehydrated, diaphanized, and included in the paraplast. The paraplast blocks were cut into 5-μm-thickness sections. Histological sections were deparaffinized and processed for immunoperoxidase. The slides were hydrated, and after being washed in PBS pH 7.2 for 5 min, the antigenic recovery was made with citrate buffer pH 6.0 for 25 min in the pressure cooker. The slides were washed in PBS and, endogenous peroxidase blockade with hydrogen peroxide and methanol was performed for 10 min in the dark. The sections were rewashed in PBS. Blocking of non-specific binding was then followed, and the slides were incubated with a blocking solution (5% skimmed milk powder in PBS) for 1 h. The sections were incubated with the primary antibody (Table 2), diluted in 1% BSA overnight in the refrigerator. After washing with PBS, the sections were exposed to the specific secondary antibody, diluted in 1% BSA, for 2 hours at room temperature. The slides were washed with PBS. The cuts were revealed with DAB (3,3′- diaminobenzidine tetrahydrochloride, Sigma - Aldrich CO^®^, United States). After successive washing with running water, the slides were counterstained with hematoxylin, dehydrated, and mounted with a coverslip using Entellan^®^. The images were obtained using the photomicroscope (Olympus BX51).

**TABLE 2:** Sequence of the primers used for RT-qPCR, designed by the company IDT.

Gene	Forward sequence	Reverse sequence
Six2	5′-GCCGAGGCCAAGGAAAGGGAG-3′	5′-GAGTGGTCTGGCGTCCCCGA-3′
c-myc	5′-AGCGTCCGAGTGCATCGACC-3′	5′-ACGTTCCAAGACGTTGTGTG-3′
c-ret	5′-GTTTCCCTGATGAGAAGAAGTG-3′	5′-GTGGACAGCAGGACAGATA-3′
Bcl-2	5′-ACGGTGGTGGAGGAACTCTT-3′	5′-GTCATCCACAGAGCGATGTTG-3′
Col-1	5′-ACCTGTGTGTTCCCCACT-3′	5′-CTTCTCCTTGGGGTTTGGGC-3′
TGFB-1	5′-GGACTCTCCACCTGCAAGAC-3′	5′-GACTGGCGAGCCTTAGTTTG-3′
Ciclin A	5′-GCC TTCACCATTCATGTGGAT-3′	5′-TTGCTGCGGGTAAAGAGACAG-3′
Bax	5′-TTCAGTGAGACAGGAGCTGG-3′	5′-GCATCTTCCTTGCCTGTGAT-3′
Bim	5′-CAATGAGACTTACACGAGGAGG-3′	5′CCAGACCAGACGGAAGATGAA-3′
Casp 3	5′-ACGGGACTTGGAAAGCATC-3′	5′-TAAGGAAGCCTGGAGCACAG-3′
GDNF	5′-CAGAGGGAAAGGTCGCAGAG-3′	5′-TCGTAGCCCAAACCCAAGTC-3′
Ki67	5′-GTCTCTTGGCACTCACAG-3′	5′-TGGTGGAGTTACTCCAGGAGAC-3′
mTOR	5′-ACGCCTGCCATACTTGAGTC-3′	5′-TGGATCTCCAGCTCTCCGAA-3′
VEGF	5′-CGGGCCTCTGAAACCATGAA-3′	5′-GCTTTCTGCTCCCCTTCTGT-3′
GAPDH	5′-CAACTCCCTCAAGATTGTCAGCAA-3′	5′-GGCATGGACTGTGGTCATGA-3′
B -catenin	5′-AGTCCTTTATGAGTGGGAGCAA-3′	5′-GTTTCAGCATCTGTGACGGTTC-3′
Map2K2	5′-ACCGGCACTCACTATCAACC-3′	5′-TTGAGCTCACCGACCTTAGC-3′
Bcl-6	5′-CCAACCTGAAGACCCACACTC-3′	5′-GCGCAGATGGCTCTTCAGAGTC-3′
PCNA	5′-TTTGAGGCACGCCTGATCC-3′	5′-GGAGACGTGAGACGAGTCCAT-3’
PRDM1	5′-CTTGTGTGGTATTGTCGGGAC-3′	5′-CACGCTGTACTCTCTCTTGG-3′
NOTCH1	5′-ACTGCCCTCTGCCCTATACA-3′	5′-GACACGGGCTTTTCACACAC-3′
IGF1	5′-AAGCCTACAAAGTCAGCTCG-3′	5′-GGTCTTGTTTCCTGCACTTC-3′
ZEB1	5′-CATTTGATTGAGCACATGCG-3′	5′-AGCGGTGATTCATGTGTTGAG-3′
ZEB2	5′-CCCTTCTGCGACATAAATACGA-3′	5′-TGTGATTCATGTGCTGCGAGT-3′

### Statistical Analysis

The Kolmogorov-Smirnov normality test with Dallal-Wilkinson-Lillefor *p*-Values was used to evaluate the Gaussian distribution of data values. The *t*-test was used, and the values were expressed as mean ± standard deviation (SD). *p* ≤ 0.05 was considered significant. GraphPad Prisma v. 01 software (GraphPad Software, Inc., United States) was used for statistical analysis and graph construction.

## Results

### Body Mass, Renal Area, and CM Cells Number

**FIGURE 1:**
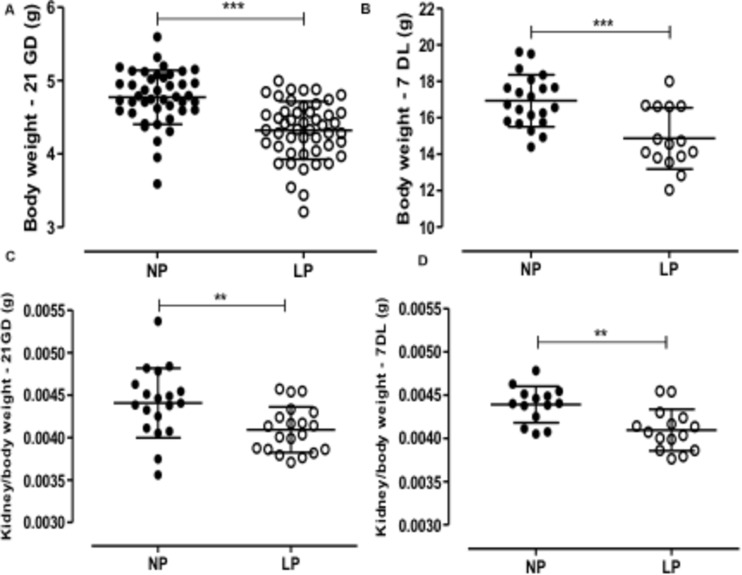
The graphics represent the male pups’ weight of body (**A**: 21GD, **B**:7DL) and kidney/body (**C**: 21GD, **D**:7DL). Mean ± SD, **p < 0.005 and ***p < 0.0001.

**FIGURE 2:**
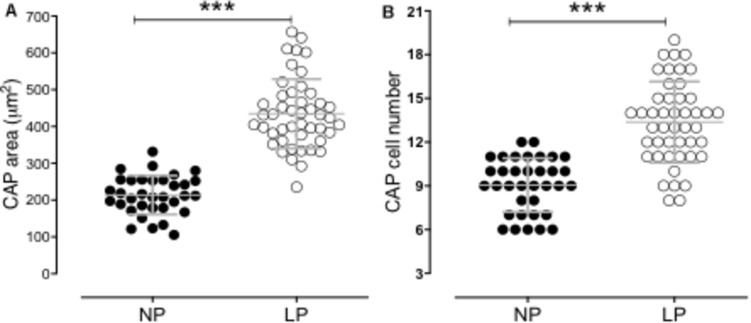
The graphics represent the CM area **(A)** and Six2 positive cells in CM **(B)**. ***p < 0.0001.

The male offspring of the LP group presented a significant reduction in the body mass in the 21GD and 7DL compared to the NP group (Figure 1). The kidney/body mass was also reduced in both 21GD and 7DL (Figure 1). In the 21GD LP animals, the nephrogenic cortical area was 31% reduced (LP = 27.5 ± 1 vs. NP = 58.1 ± 1.6, *n* = 4 of each, p < 0.0001) and the medullar 34% enhanced (LP = 72.5 ± 1 vs. NP = 42 ± 1.6, *n* = 4 of each, p < 0.0001) when compared to that observed in NP group. The CMs presented an increase in both areas (103%) and several Six2 positive cells (32%) in the 21GD LP kidneys (Figure 2). In the 7DL LP animals, the renal areas, as well as the percentage of the two renal areas were not altered (cortical: LP = 56 ± 3 vs. 69 ± 9, *n* = 4; medullar: LP = 44 ± 3 vs. NP = 39 ± 2, *n* = 4).

### Expression of miRNAs by miRNA-Seq

By expressing a global miRNA profiling analysis, we founded 21 differently expressed miRNAs, of which 12 were upregulated and 9 downregulated in 21-GD, LP fetuses, compared to age-matched NP offspring (Table 3). In 7-DL LP offspring, there were 74 differentially expressed miRNAs, 46 were upregulated, and 28 were downregulated (Table 3). The top expressed miRNAs and their functions, pathways, and networks were identified using Ingenuity Software (Table 4). After obtaining a list of differential expressed miRNAs, the software Ingenuity Pathways Analysis (IPA) was used to determine the functions, pathways, and networks involved with these differentially expressed miRNAs. In this analysis, an experimental target is used, instead of a putative one, as it is more reliable. Having obtained these pathways, we analyzed those that we were interested in concerning the processes of cell proliferation, apoptosis, and differentiation. We then selected, from the top 10, the miRNAs that were involved in these pathways. Afterwards, we also checked the miRTarBase database, which shows target microRNA interactions. Cross-referencing the data from Ingenuity and mirTarBase, we selected the target miRNAs and genes that we would validate by RT-PCR.

**TABLE 3:** Lists of the deregulated miRNAs obtained by miRNA-Seq.

miRNAs up-regulated – 21 DG	FC	miRNAs downregulated – 21 DG	FC
25_TACCCTGTAGATCCTAATTTGT_rno-mir-10b	1.92	7_TGAGGTAGTAGATTGTATAGT_rno-let-7f-2	0.72
53_TGTGCAAATCCATGCAAAACTG_rno-mir-19b-1	1.56	52_TCAGTGCATGACAGAACT_rno-mir-152	0.63
14_TCTGGCTCCGTGTCTTCACTCC_rno-mir-149	1.72	rno-let-7f-5p	0.80
rno-miR-326-3p	1.70	5_TGAGGTAGTAGTTTGTGCTGTTA_rno-let-7i	0.78
rno-miR-615	1.67	rno-let-7g-5p	0.85
10_GGCAGAGGAGGGCTGTTCTTC_rno-mir-298	1.53	rno-miR-98-5p	0.83
15_TAGGTAGTTTCCTGTTGTTGGGT_rno-mir-196b-2	1.44	rno-miR-103-3p	0.79
56_TCGGATCCGTCTGAGCTTGGC_rno-mir-127	1.57	rno-miR-451-5p	0.63
rno-let-7e-3p	1.56	15_TAGGTAGTTTCCTGTTGTTGGGAT_rno-mir-196b-2	0.64
rno-miR-127-3p	1.48		
10_TGAGGGGCAGAGAGCGAGACTTT_rno-mir-423	1.39		
51_AGCAGCATTGTACAGGGCTATGT_rno-mir-103-1	1.57		

**miRNAs up-regulated – 7 DL**	**FC**	**miRNAs downregulated – 7 DL**	**FC**

50_CTAGACTGAGGCTCCTTGAGGA_rno-mir-151	1.80	25_TACCCTGTAGATCCGAATTCGT_rno-mir-10b	0.41
69_TACAGCAGGCACAGACAGGCAG_rno-mir-214	2.43	60_TCAGTGCACTACAGAACTTTGT_rno-mir-148a	0.64
rno-miR-298-5p	1.94	59_TGAGATGAAGCACTGTAGCTCTT_rno-mir-143	0.72
15_TCCCTGAGGAGCCCTTTGAGCCTG_rno-mir-351-1	1.70	25_TACCCTGTAGATCCTAATTTGT_rno-mir-10b	0.43
rno-miR-1249	2.4	59_TGAGATGAAGCACTGTAGCTCT_rno-mir-143	0.72
47_TATTGCACTTGTCCCGGCCTGTAA_rno-mir-92a-1	1.64	rno-miR-199a-3p	0.73
15_TCCCTGAGGAGCCCTTTGAGCCTG_rno-mir-351-2	1.78	rno-miR-26b-5p	0.63
8_AGACCCTGGTCTGCACTCTGTCT_rno-mir-504	2.33	rno-miR-143-3p	0.74
11_TGTAAACATCCCCGACTGGAAGCT_rno-mir-30d	1.59	15_TAGGTAGTTTCCTGTTGTTGGGT_rno-mir-196b-2	0.53
14_AACATTCAACGCTGTCGGTGAGTT_rno-mir-181a-1	1.39	5_TGAGGTAGTAGTTTGTGCTGTTAT_rno-let-7i	0.48
14_AACATTCATTGCTGTCGGTGGGTT_rno-mir-181b-2	1.53	rno-let-7a-5p	0.69
rno-miR-181a-5p	1.28	22_AAAGTTCTGAGACACTCTGACT_rno-mir-148a	0.64
47_TATTGCACTTGTCCCGGCCTGTAAA_rno-mir-92a-1	1.69	rno-miR-148a-3p	0.51
61_AGCTACATCTGGCTACTGGGTCTCT_rno-mir-222	1.84	59_TGAGATGAAGCACTGTAGCTC_rno-mir-143	0.73
rno-miR-125a-5p	1.70	15_TCTTTGGTTATCTAGCTGTAT_rno-mir-9a-2	0.54
rno-miR-486	1.77	25_TACCCTGTAGAACCGAATTTGTGTGT_rno-mir-10b	0.55
47_CTGGCCCTCTCTGCCCTTCCGTTT_rno-mir-328a	2.13	rno-miR-196b-5p	0.69
15_TCCCTGAGGAGCCCTTTGAGCCT_rno-mir-351-1	1.69	14_TGAGGTAGGAGGTTGTATAGT_rno-let-7e	0.67
11_TGTAAACATCCCCGACTGGAAGC_rno-mir-30d	1.58	rno-miR-17-1-3p	0.60
6_CACCCGTAGAACCGACCTTGCGA_rno-mir-99b	1.62	15_TAGGTAGTTTCCTGTTGTTGGGT_rno-mir-196b-1	0.57
47_TATTGCACTTGTCCCGGCCTGTTAT_rno-mir-92a-1	1.68	22_CAGCAGCAATTCATGTTTTGGAT_rno-mir-322-1	0.60
69_TACAGCAGGCACAGACAGGCAGT_rno-mir-214	1.67	24_TAGGTAGTTTCATGTTGTTGGGT_rno-mir-196a	0.64
32_TCCTTCATTCCACCGGAGTCTG_rno-mir-205	1.88	21_TACCCTGTAGAATCGAATTTGT_rno-mir-10a	0.57
48_TACTAGACTGAGGCTCCTTGAG_rno-mir-151	1.92	48_TGTGACAGATTGATAACTGAAAGT_rno-mir-542-3	0.60
70_ACAGCAGGCACAGACAGGCAGT_rno-mir-214	1.66	15_TCTTTGGTTATCTAGCTGTATG_rno-mir-9a-1	0.57
14_TCCCTGAGACCCTTTAACCTGTG_rno-mir-125a	1.64	rno-miR-146b-5p	0.66
rno-miR-151-3p	1.60	5_TGAGGTAGTAGTTTGTGCTG_rno-let-7i	0.57
14_TCCCTGAGACCCTTTAACCTG_rno-mir-125a	1.64	21_TACCCTGTAGAACCGAATTTGA_rno-mir-10a	0.61
14_TCCCTGAGACCCTTTAACCTGTGG_rno-mir-125a	1.65	14_TTCAAGTAATTCAGGATAGGTT_rno-mir-26b	0.79
rno-miR-125b-5p	1.48		
16_TGTAAACATCCTACACTCTCAGCT_rno-mir-30c-1	1.17		
11_TGTAAACATCCCCGACTGGA_rno-mir-30d	1.39		
46_AGCTCGGTCTGAGGCCCCTCAGA_rno-mir-423	1.87		
45_TCAGGCTCAGTCCCCTCCCGATT_rno-mir-484	1.86		
54_TATTGCACTTGTCCCGGCCTGAAA_rno-mir-92a-2	1.80		
3_TCCTGTACTGAGCTGCCCCGA_rno-mir-486	1.64		
45_ATCACATTGCCAGGGATTTCCAA_rno-mir-23a	1.80		
24_AAGTTCTGTTATACACTCAGGCT_rno-mir-148b	1.73		
60_CTATACAACCTACTGCCTTCCT_rno-let-7b	1.78		
15_TCCCTGAGGAGCCCTTTGAGCC_rno-mir-351-1	1.52		
10_GGCAGAGGAGGGCTGTTCTTCC_rno-mir-298	1.56		
15_TCCCTGAGGAGCCCTTTGAGCCT_rno-mir-351-2	1.50		
15_TCCCTGAGGAGCCCTTTGAGCCTGT_rno-mir-351-1	1.54		
rno-miR-149-5p	1.73		
14_AACATTCAACGCTGTCGGTGAG_rno-mir-181a-1	1.22		

**TABLE 4:** Top canonical pathways affected by differentially expressed miRNAs in 21 DG and 7 DL metanephros.

21 GD	Pathway analysis results (IPA)	Number of miRNAs	*p*-Value/score
**NP vs LP**	**Top Molecular and Cellular Functions**		
	Cellular Development	64	4.77E-02 – 5.79E-14
	Cellular Growth and Proliferation	64	4.43E-02 – 5.79E-14
	Cellular Movement	36	4.77E-02 – 1.07E-08
	Cell Cycle	23	4.76E-02 – 1.65E-06
	Cell Death and Survival	38	4.29E-02 – 5.29E-05
	**Top Networks**		
	Cancer, Organismal Injury and Abnormalities, Reproductive System Disease		54
	**Top Tox Lists**		
	Renal Ischemia-Reperfusion Injury microRNA Biomarker Panel (Mouse) **Top 10 highly expressed miRNAs** miR-10b-5p; miR-148a-3p; miR-330-5p; miR-615-5p let-7a-5p; miR-298-5p; miR-127-3p; let-7e-3p miR-103-3p; miR-423-3p		2.88E-17

**7 DL**	**Pathway analysis results (IPA)**	**Number of miRNAs**	***p*-Value/score**

**NP vs LP**	**Top Molecular and Cellular Functions**		
	Cellular Development	18	4.71E-02 – 4.76E-08
	Cellular Growth and Proliferation	15	3.44E-02 – 4.76E-08
	Cell Cycle	07	3.98E-02 – 5.81E-06
	Cellular Movement	11	4.83E-02 – 5.81E-06
	Cell Death and Survival	13	4.50E-02 – 6.10E-05
	**Top Networks**		
	Cancer, Gastrointestinal Disease, Organismal Injury and Abnormalities		28
	**Top Tox Lists**		
	Renal Ischemia-Reperfusion Injury microRNA Biomarker Panel (Mouse) **Top 10 highly expressed miRNAs** miR-10b-5p; miR-214-3p; miR-298-5p; miR-143-3p miR-1249-3p; miR-504-5p; miR30c-5p; miR-181a-5p miR-199a-5p; miR-221-3p		1.01E-07

### Validation of miRNA Expression

**FIGURE 3:**
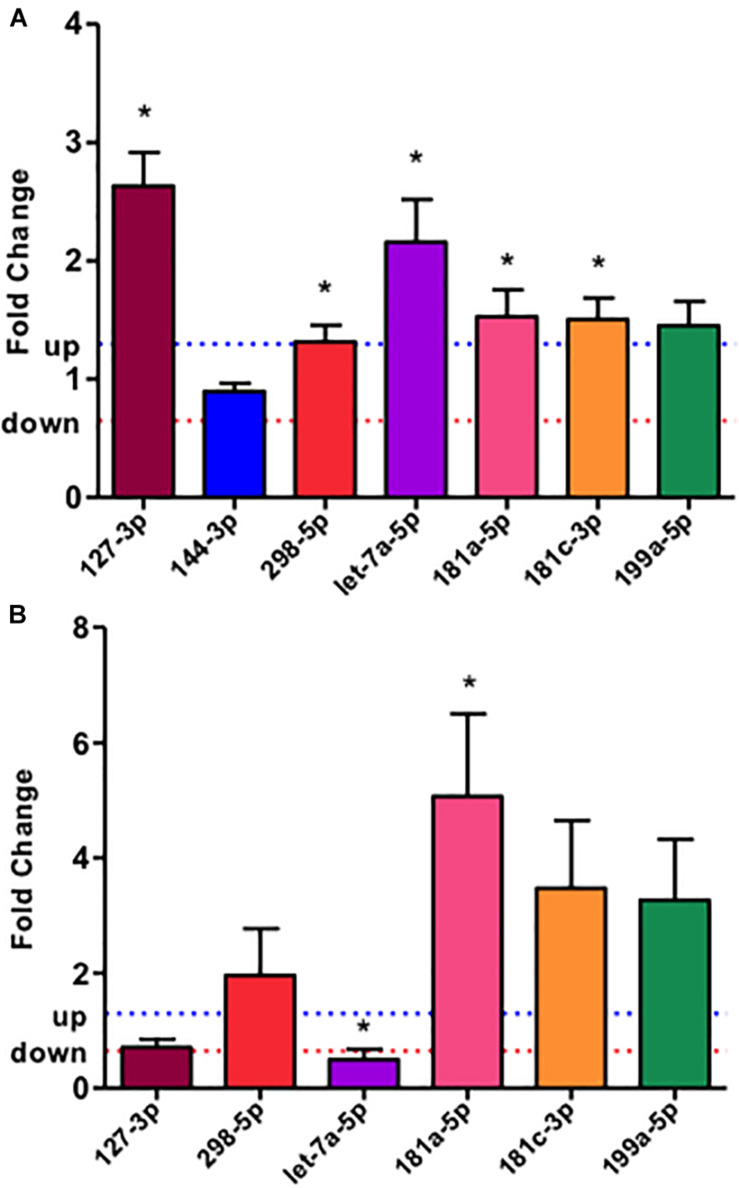
Expression of miRNAs in the LP kidneys from 21 GD **(A)** and 7DL **(B)** rats compared to the level of expression in the NP group. The expression of each miRNA was normalized with U6 and U87. The cutoff point variation of 1.3 (upwards) or 0.65 (downwards) was established, and data are expressed as fold change (mean ± SD, *n* = 4) concerning the control group. *p ≤ 0.05: statistical significance versus NP.

*In* the animals of 21GD from the LP group, miR-127-3p, miR-298-5p, let-7a-5p, miR-181a-5p, and miR-181c-3p were upregulated in the kidneys compared to NP animals. The results do not show any difference in miR-144-3p and miR-199a-5p expression, comparing both groups (Figure 3A). Only miR-181a-5p was upregulated, and let-7a-5p was downregulated *in* the kidneys from 7DL LP compared to age-matched NP offspring (Figure 3B). Table 5 and additional data showed in the Supplementary Material file revealed the values obtained by miRNAs sequencing with the RT-qPCR validation data. Although significant miRNA expression difference was observed in LP relative to NP offspring, the fold change (FC) of the validated miRNAs was similar to both techniques.

**TABLE 5:** Comparison between the values obtained in the miRNA sequencing and the validation by RT-qPCR.

miRNA (21 GD)	RNA-Seq logFC	Fold Change	*p-Value*	miRNA (21GD)	Fold Change	qPCR logFC	*p-Value*
miR-127-3p	0.565173065	1.4796	0.04442707	miR-127-3p	2.6333	1.3969	6.02E-03
miR-144-3p				miR-144-3p	0.8974	−0.1562	0.1723848
miR-298-5p	0.613353841	1.5298	0.03299593	miR-298-5p	1.3170	0.3972	0.0402289
let-7a-5p				let-7a-5p	2.1660	1.1150	0.0059875
miR-181a-5p				miR-181a-5p	1.5286	0.6122	0.0421524
miR-181c-3p				miR-181c-3p	1.5058	0.5906	0.0228443
miR-199a-5p				miR-199a-5p	1.4513	0.5373	0.0604275

**miRNA (7 DL)**	**RNA-Seq logFC**	**Fold Change**	***p-value***	**miRNA (7 DL)**	**Fold Change**	**qPCR logFC**	***p-value***

miR-127-3p				miR-127-3p	0.730578	−0.4529	6.01E-02
miR-144-3p				miR-144-3p	No expressed		
miR-298-5p	0.958238445	1.9429	0.00055761	miR-298-5p	1.9583	0.9696	0.3025506
let-7a-5p	−0.537048101	0.6892	0.01533047	let-7a-5p	0.4997	−1.0008	0.0269524
miR-181a-5p	0.357437752	1.2811	0.00498333	miR-181a-5p	5.0680	2.3414	0.0217739
miR-181c-3p				miR-181c-3p	3.4746	1.7968	0.0731242
miR-199a-5p				miR-199a-5p	3.2639	1.7066	0.0858642

### miRNA Targets and ZEB1/2 Expression

**FIGURE 4:**
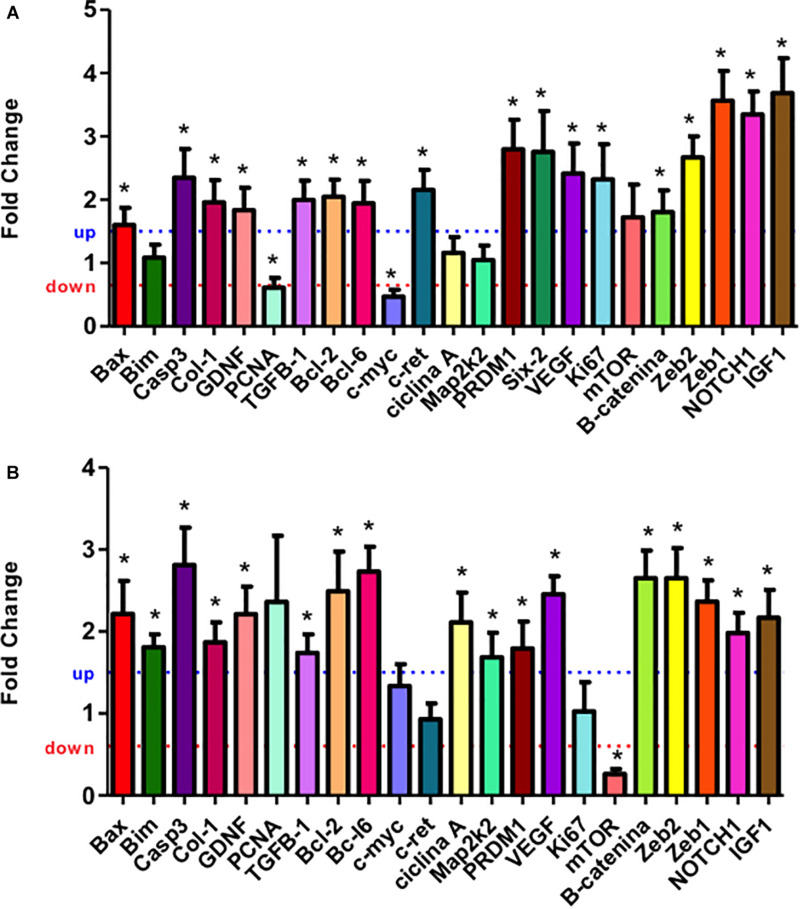
Renal expression estimated by SyBR green RT-qPCR of mRNA from 21GD **(A)** to 7DL **(B)** LP rats. The expression was normalized with GAPDH. The authors established a cutoff point variation of 1.3 (upwards) or 0.65 (downwards), and data are expressed as fold change (mean ± SD, *n* = 4) concerning the control group. *p ≤ 0.05: statistical significance versus NP.

The mRNA expression of the most elected predicted targets of miRNAs and ZEB1/2 were enhanced in 21GD and 7DL kidneys LP compared to NP. Thus, Bax, Caspase-3, GDNF, Collagen 1, TGFβ, Bcl-2, Bcl-6, PRDM1, β-catenin, and IGF1. At 7DV, Bim, cyclin A, and Map2k2 mRNA expression were enhanced, and mTOR was reduced in LP. At 21GD, PCNA and c-myc mRNA expression was downregulated (Figure 4) in maternal protein-restricted offspring. Using results obtained in the 17GD metanephros using the same model presented here (Sene et al., 2021) and 21GD and 7DL kidneys, we have traced curves where we can observe the more detailed and comparative view of mRNAs expression control by microRNAs (Figure 4).

### Immunohistochemistry

**FIGURE 5:**
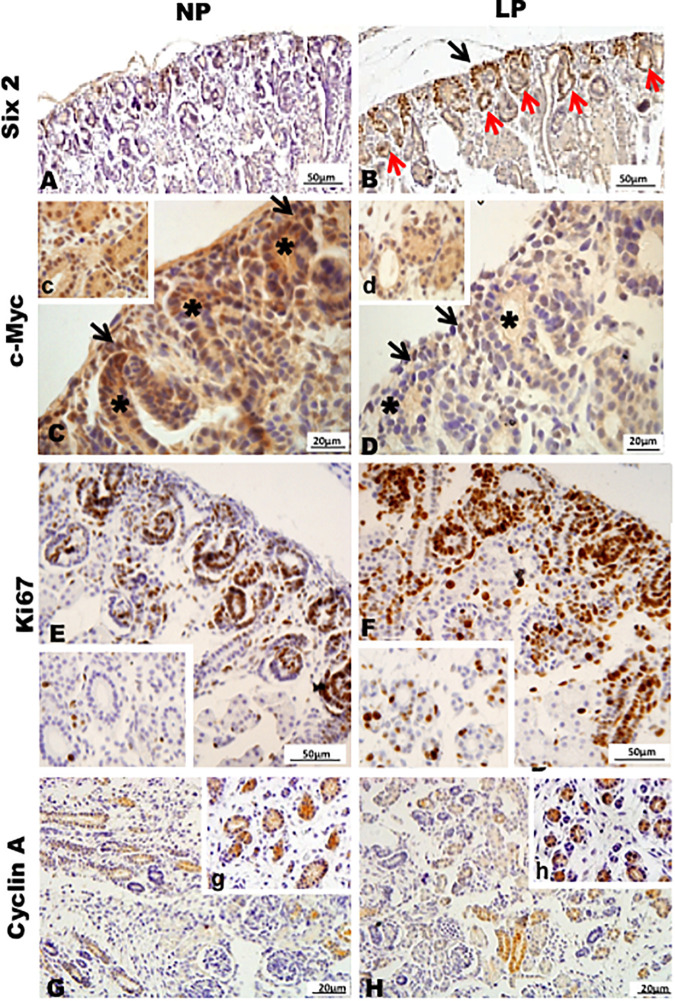
Immunoperoxidase in kidneys of 21GD rats for **(A,B)** Six 2, (C,D) c-Myc, **(E,F)** Ki67, and **(G,H)** cyclin A for the NP **(A,C,E,G)** and LP **(B,D,F,H)** groups. CM (black arrow); UB (*) renal structures in differentiation (red arrow). The inserts represent a medullar area.

**FIGURE 6:**
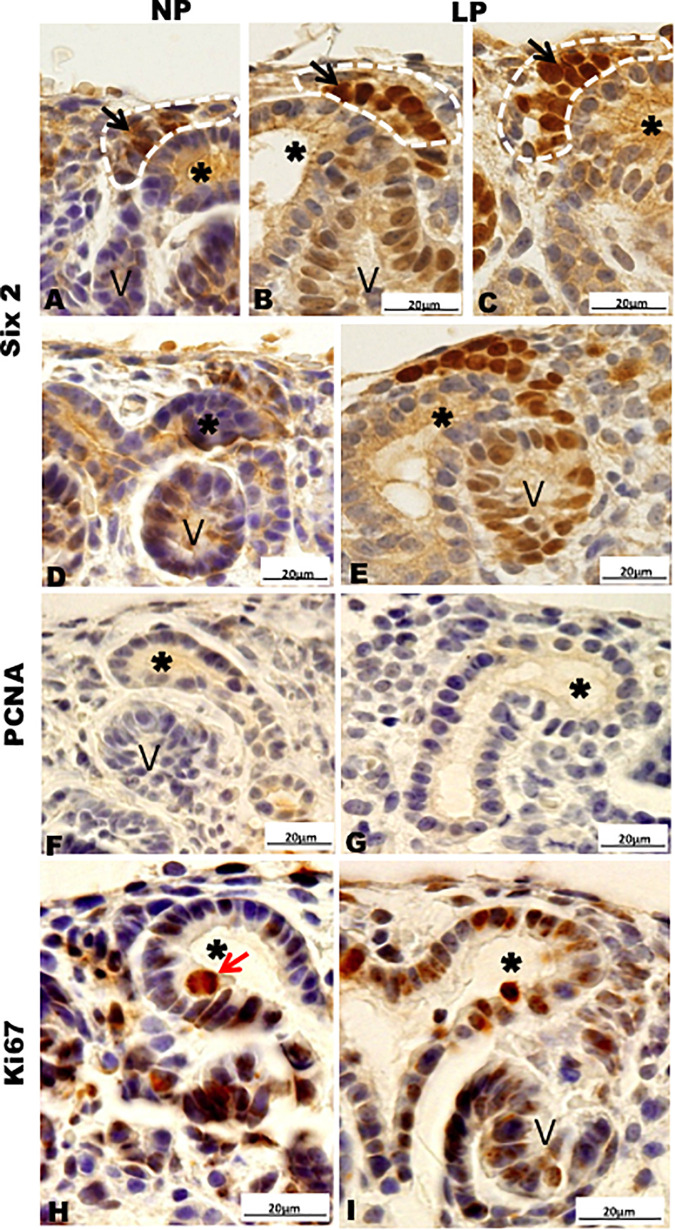
Immunoperoxidase in kidneys of 21GD rats for **(A–E)** Six 2, **(F,G)** PCNA, **(H,I)** Ki67 for the NP **(A,B,D,F,H)** and LP **(C,E,G,I)** groups. CM (black arrow); UB (*) renal vesicles (V); cell in late anaphase (red arrow).

**FIGURE 7:**
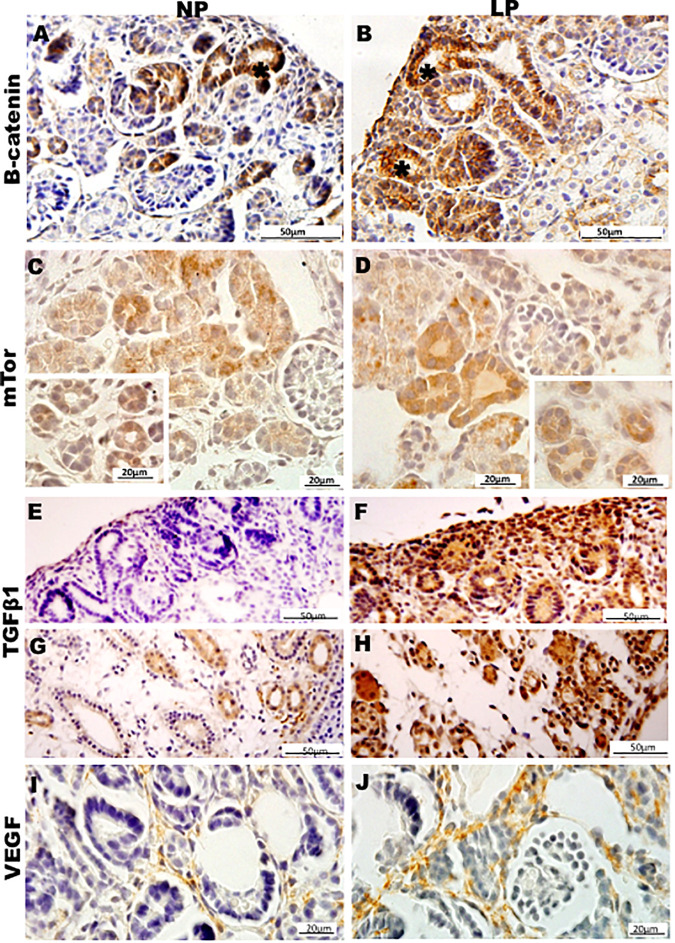
Immunoperoxidase in kidneys of 21GD rats for **(A,B)** B-catenin, **(C,D)** mTor, **(E–H)** TGFb1, and **(I,J)** VEGF for the NP **(A,C,E,G,I)** and LP **(B,D,F,H,J)** groups. The inserts represent the medullar area. UB (*) renal vesicles.

**FIGURE 8:**
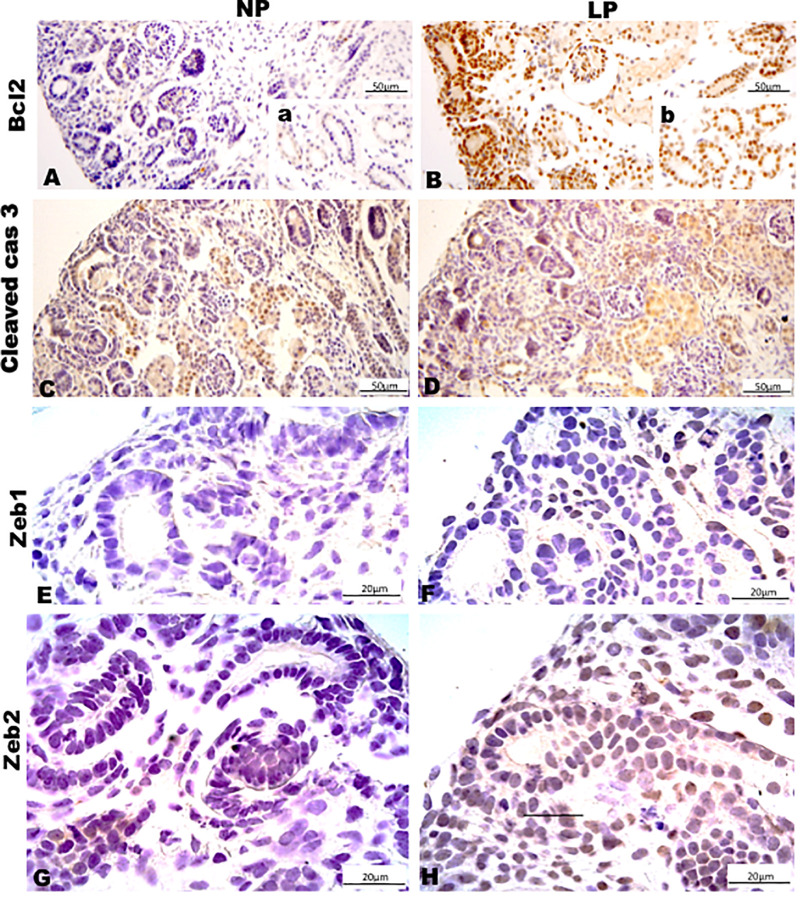
Immunoperoxidase in kidneys of 21GD rats for **(A,B)** Bcl2, **(C,D)** cleaved cas 3, **(E,F)** Zeb1, and **(G,H)** Zeb2 for the NP **(A,C,E,G)** and LP **(B,D,F,H)** groups. The inserts represent the medullar area.

Results in 21GD: In a general view of Six2 labeled LP kidney, we verified more intensive reactivity and more cells labeled in comparison whit that observed in NP. These labeled cells are present in both CM and differentiated LP structures and not in NP (Figures 5A,B). In a more detailed view, we can observe that the more intensive Six2 reactivity is present in CM cells whose number is raised in LP (Figures 6A-E). In a more reduced pattern, Six2-positive cells are migrating from CM and present in differentiating vesicles only in the LP kidney (Figures 6B,E). We can also see that all cells are more extensive than those present in NP and enlarged nuclei with loose chromatin and evident nucleolus (typical of interphases’ nuclei). Simultaneously, in NP, we can observe more intense hematoxylin-labeled nuclei, indicating condensed chromatin (Figures 6A-E). The cMyc reactivity was remarkably reduced in the nephrogenic zone but not so expressive in the medulla in LP compared to NP (Figures 6C,D). Already, LP kidneys presented much more Ki67 reactive cells than NP in all tissue (Figures 5E,F). In the highest magnification, we can see Ki67-positive cells, and in LP, the majority have their nucleoli evident, whereas, in NP, this is not visible (Figures 6H,I). The cyclin A reactivity was the same in NP and LP kidneys and was only found in tubular segments below the nephrogenic zone (Figures 5G,H) being absent in this zone (Figures 6F,G). In the highest magnification, we observed that in LP kidneys, the UB epithelial cells have round nuclei, representing their cubic shape, while in NP, we can keep elongated nuclei, characteristic of cylindrical epithelia (Figure 6). By the immunolocalization of β-catenin, we observe that this protein was present in many more LP kidney structures than observed in NP (Figures 7A,B). mTor was present in tubular segments below the nephrogenic zone and was increased in LP (Figures 7C,D). The intensity of TGFβ1 reactivity was significantly enhanced in all renal tissue in LP than in NP (Figures 7E-H). The VEGF was more intensive and present in the higher area of extracellular matrix in LP than in NP (Figures 7I,J). All cells of LP kidneys presented the anti-apoptotic Bcl2 protein, and in NP, this protein was present in a low number of cells (Figures 8A,B). Activated caspase-3 distribution was not different from NP and LP kidneys (Figures 8C,D). Zeb 1 and 2 were present in a higher number of cells from a nephrogenic LP zone than NP (Figure 8).

**FIGURE 9:**
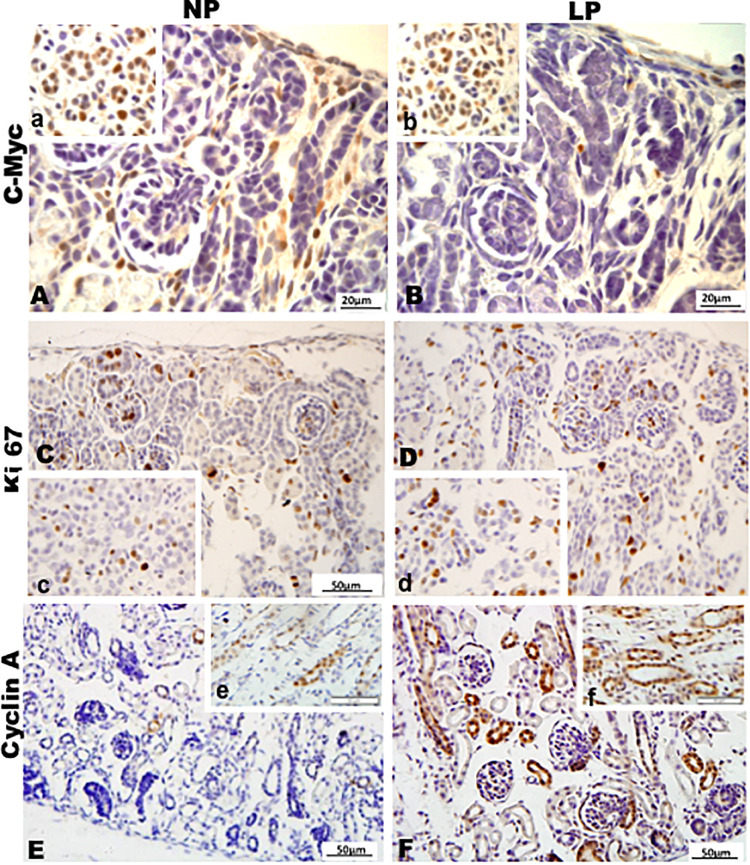
Immunoperoxidase in kidneys of 7DL rats for **(A,B)** c-Myc, **(C,D)** Ki67, and **(E,F)** cyclin A for the NP **(A,C,E)** and LP **(B,D,F)** groups. The inserts represent the medullar area.

**FIGURE 10:**
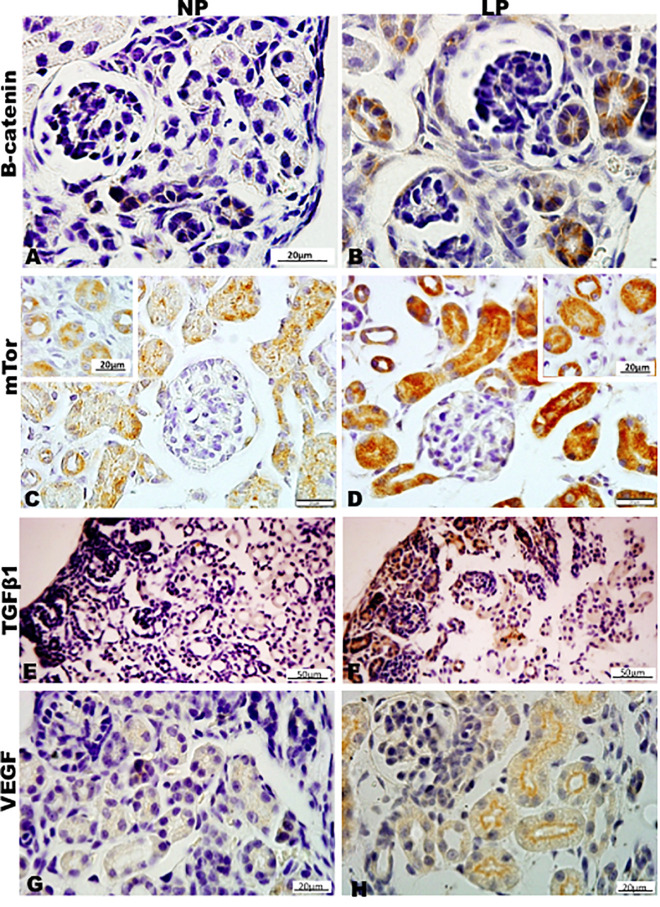
Immunoperoxidase in kidneys of 7DL rats for **(A,B)** B-catenin, **(C,D)** mTor, **(E,F)** TGFb1, and **(G,H)** VEGF for the NP **(A,C,E,G)** and LP **(B,D,F,H)** groups. The inserts represent the medullar area.

**FIGURE 11:**
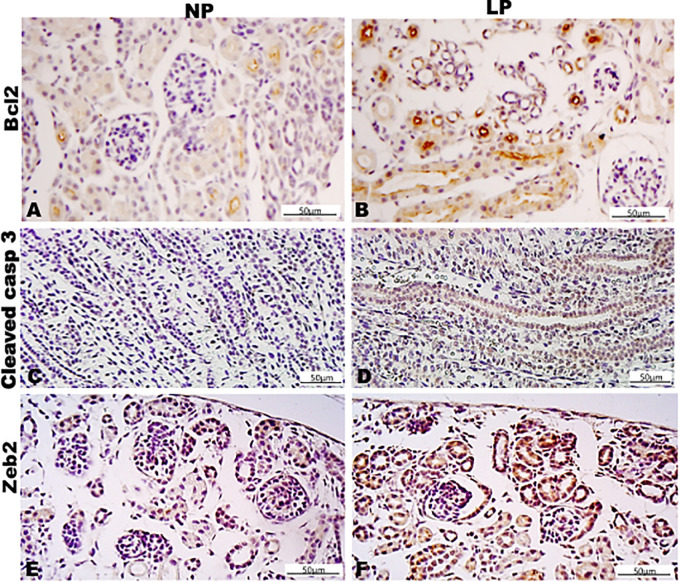
Immunoperoxidase in kidneys of 7DL rats for **(A,B)** Bcl2, **(C,D)** cleaved casp 3, and **(E,F)** Zeb2 for the NP **(A,C,E)** and LP **(B,D,F)** groups.

By the immunoperoxidase, the data has shown that c-Myc positive cells were more numerous in the extracellular matrix of the 7-DL NP nephrogenic zone when compared to LP. In the medulla, the tubular cells are labeled in a similar pattern in both groups (Figures 9A,B). Although not so apparent, the number of Ki67 positive cells in LP was higher than in NP (Figures 9C,D). Cyclin A was intensely increased in all renal tissue from LP than that viewed in NP (Figures 9E,F). Elevated β-catenin and mTor reactivity were obtained in tubular segments of LP renal tissue compared to NP (Figures 10A-D). TGF-β1 was increased, more prominent in the outer cortex of LP than NP (Figures 10E,F). LP kidneys have elevated VEGF immunoreactivity in cytosolic and apical tubular cells’ compartments (Figures 10G,H). Bcl2 was more intensely labeled and distributed in the tubular cells of LP, and cleaved caspase-3 reactivity was raised in the medulla of LP (Figures 11A-H). LP kidneys presented a much higher number of cells showing Zeb 2 reactivity than NP (Figures 11I,J).

## Discussion

During perinatal kidney development in rats, nephrogenesis depends on adequate progenitor cells’ self-renewal, survival, and differentiation capacity. Newborn rats maintain active nephrogenesis. There exists a consensus that miRNAs drive renal development regulating gene expression of proteins involved in key signaling pathways (Lv et al., 2014; Marrone and Ho, 2014; Wei et al., 2014; Phua et al., 2015). Recently, we established miRNA and elected target mRNA expression in maternal low-protein intake 17GD male metanephros (Sene et al., 2021). In the current study, we performed a similar evaluation, ensuring UB branching and mesenchymal to epithelial transformation in 21GD fetus and 7DL kidneys from LP progeny compared to NP offspring. Thus, we depicted a time-course curve of most altered miRNAs and elected mRNAs transcripts during nephrogenesis in the maternal low protein intake model by data from these ages. This observation aims to elucidate particular pathways related to the reduced nephron number observed in this model (Mesquita et al., 2010a, b).

Surprisingly, as the discussion below, the present study data showed some discrepancies between miRNAs and their predicted targets’ expected expression.

These findings confirm prior studies demonstrate that miRNAs could act as positive transcription factors (Vasudevan et al., 2007; Zhang et al., 2014). Furthermore, the algorithms could not find all miRNA-gene interactions, and it has been demonstrated that they detect 60% of all available targets (Reczko et al., 2012; Vlachos and Hatzigeorgiou, 2013).

The present data found 12 upregulated and 9 downregulated miRNAs in 21GD, and 46 were upregulated and 28 downregulated in 7DL LP progeny compared to age-matched NP offspring kidneys in the present study. The current data identified the top 10 deregulated miRNAs in 17 GD and 21 GD fetuses and 7DL offspring. Here, we have selected 7 miRNAs involved in the protein synthesis that are key regulators during renal ontogenesis.

The miR-181a has been related to the critical developmental process of cell proliferation, migration, and apoptosis (Chen et al., 2010). The current study showed an increased expression of miR-181a-5p in 21GD and 7DL LP rats relative to age-matched NP offspring. In the 21GD, both Bcl2 and caspase-3 mRNA are also enhanced, but as a previous study shows, Bcl2 inhibits Caspase-3 activation, reducing cellular apoptosis (Scorrano and Korsmeyer, 2003).

The present study shows an enhanced Bcl2 reactivity in 21GD LP progeny by immunohistochemistry compared to age-matched NL offspring. However, the cleaved Caspase-3 was not altered. Thus, although overexpressed miR-181a has been related to decreased Bcl2 protein levels (Li et al., 2016), here, we do not find an undoubted association.

In our model, the Bcl2 mRNA was not modulated by miR-181a-5p, considering the depicted expression curve. Also, TGFβ1 mRNA, another known predicted target of miR-181a-5p, no observed association was identified in the present study.

GDNF-RET signaling is the primary regulator of the ureteric bud branching process (Schuchardt et al., 1994; Moore et al., 1996; Pichel et al., 1996; Sanchez et al., 1996), and both GDNF and c-ret mRNA are described how the target of miR-181a-5p. Here, the results showed a negative and reciprocal relationship between miR-181a-5p and c-ret mRNA expression. By the curve of expression, GDNF mRNA was not modulated by miR-181a-5p but, GDNF and c-ret mRNA were upregulated in LP at 21GD fetus, indicating an active induction of UB ramification. In the 7DL, the LP progeny kidneys also showed an enhanced GDNF expression but associated with decreased c-ret mRNA expression, indicating a reduction in the UB ramification in this age.

During kidney development in LP 17GD offspring, we have demonstrated that miR-181c enhanced the expression of Six2 and negatively regulates cell proliferation (Sene et al., 2021), confirming results by Lv et al. (2014).

The present results demonstrated in 21GD LP kidney, upregulation of ki67 and Six2 mRNAs in parallel with raised miR-181c-3p expression. In the 7DL, the miR-181c-3p expression was enhanced in LP compared to the NP offspring kidney, which is compatible with the reduced Six2 expression in this age.

**FIGURE 12:**
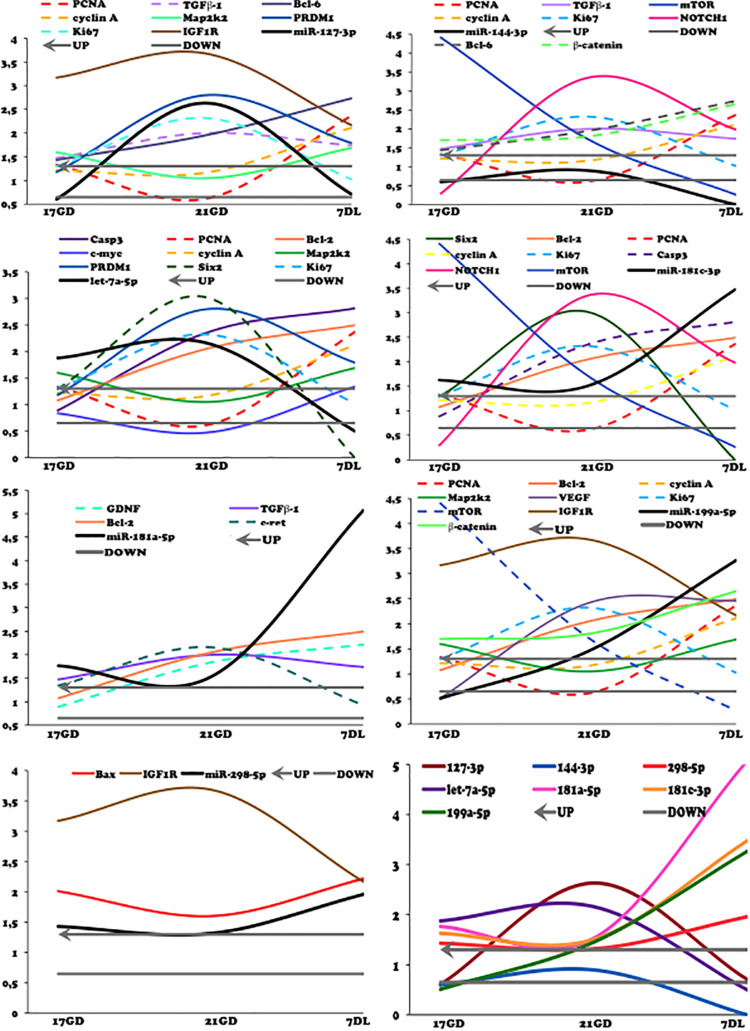
Curves of validated miRNAs and their predicted target mRNAs obtained by miRTarBase and Ingenuity (solid lines) and from literature (dashed line). The expression was normalized with GAPD. The authors established a cutoff point variation of 1.3 (upwards) or 0.65 (downwards), and data are expressed as fold change (mean) concerning the control group.

As demonstrated in Figure 12, the variations in miR-181c-3p expression were not causally associated with results observed to Bcl2, casp3, NOTCH1, and mTOR mRNA in the LP relative NP group at the same age. Concerning proliferation, we observed that miR-181c-3p negatively regulated ki67 and positively cyclin-A and PCNA mRNAs in LP kidneys. The present study has shown unexpected discrepancies concerning cell cycle markers; in such a way that ki67 mRNA expression and protein reactivity were significantly upregulated; however, PCNA was unaltered. Cyclin-A mRNA and protein were reduced in 21GD kidneys LP when compared to age-matched NP progeny. Considering 21GD kidneys in NP fetus, an enhanced ki67 reactivity was observed in developing nephrons, but, unprecedently, cyclin-A immunoreactivity was not present. In this way, the cyclin-A reactivity was only present in tubular segments below the nephrogenic zone.

The upregulation of miR-127 has been related to increased cell proliferation (Pan et al., 2012). As we observed here, the ki67 mRNA expression and protein reactivity were elevated in the nephrogenic zone of maternal protein-restricted offspring kidney. The CAP area and the number of Six 2 expressing cells in the CAPs were also elevated (103 and 32%, respectively) in 21GD LP kidneys.

The depicted curves of miR and mRNAs expression in the different studied ages showed that miRNAs 127-3p, 144-3p, and Let7a-5p regulated positively Ki67 and negatively cyclin-A and PCNA mRNA expression. Conversely, such as mentioned anteriorly, miR-181c-3p downregulated ki67 associated with increased cyclin-A and PCNA mRNAs expression in LP kidneys.

Unprecedentedly, in 21GD LP kidneys, the UB epithelial cells have round nuclei, representing their cubic shape, with loose chromatin and evident nucleolus (typical of interphases’ nuclei). Simultaneously, in 21GD NP, we found elongated nuclei, characteristic of cylindrical epithelia, with dense chromatin (Figure 7). The CM and disperse mesenchyme cell nuclei have the same aspects observed in UB in both NP and LP at a similar age.

Despite raised ki67 expression in the nephrogenic zone in the 21GD LP, the nuclear characteristics described above are incompatible with cells in the mitosis process. Thus, we cannot exclude a possible cell-cycle arrest in these structures. However, Ki67 is expressed throughout the cell cycle, including G1, and some non-proliferative cells can poise at G1 for an extended period without cell division (Alvarez et al., 2019).

IGF-1R is a plasma membrane receptor with tyrosine kinase activity, whose inhibitors have been used to treat carcinoma cell lines, with a strong non-proliferative effect (Fuentes-Baile et al., 2020). The IGF-1R elevated expression has also been associated with inhibition of apoptosis and increased proliferation rate and angiogenesis in patients with cancer (Matsubara et al., 2009; Valsecchi et al., 2012). The upregulation of miR 127-3p in the 21GD kidneys from LP animals was accompanied by enhanced IGF1 expression. The rise of ki67 and the decrease of activated caspase-3 in the 21GD LP nephrogenic zone coincide with the activities described previously. The study also showed that miR 127-3p negatively modulated map2k2 mRNA expression, which is in direct contrast to the Ki67 expression.

We may infer that enhanced Ki67 and Bcl-6 mRNA expression in the 17GD LP mesenchymal metanephros compared to age-matched NP progeny is also associated with miR-127-3p downregulation expression. These results may be related to the cell proliferative process (Sene et al., 2021). We found an increased miR-127-3p expression in the 21GD LP kidneys accompanied by enhanced Ki67 expression compared to 17DG LP. Thus, we may suppose that counter-regulatory mechanisms acted by other pathways preventing cell proliferation. At 7DL progeny, the miR-127-3p and Ki67 mRNA encodings in the LP kidneys were reduced compared to 21GD, and the Ki67 immunoreactivity was remarkably like NP offspring kidneys.

A previous study using human cancer cell culture incubated in propidium iodide found a reduction in the proportion of cells in the G2/M phase of the cycle and consequent suppression of renal carcinoma proliferation induced by enhanced miR-144 (Xiang et al., 2016). These authors suggest S/G2 cell cycle arrest. Here the miR-144-3p expression was unaltered in 21GD LP relative to NP; however, its curve of expression showed a positive control of Ki67 associated with the reduced encoding of cyclin A and PCNA. The current study was not able to detect the miR-144-3p expression at 7DL kidneys. In prior research, the authors demonstrated mTOR inhibition by miR-144 overexpression (Xiang et al., 2016). Here, the miR-144-3p expression does not influence mTOR expression.

Recently, Sun et al. (2020) demonstrated that miR-144-3p inhibits cell proliferation and delays the G1/S phase transition of colorectal cancer cells. Additionally, the authors determined Bcl6 as a new target of miR-144-3p, suggesting that miR-144-3p/Bcl6 repressed cell proliferation possibly through Wnt/β-catenin signaling.

They found that miR-144-3p downregulated the expression of β-catenin, c-myc, and cyclin D1 (Sun et al., 2020). In the present study, the Bcl6, β-catenin, and miR-144-3p analysis depicted curves of expression in LP revealed remarkably similar curves negatively regulated by their miR.

In this way, the results showing unaltered cyclin-A and Map2k2 in the 21GD LP kidneys and downregulated PCNA mRNAs may be associated with reducing mitosis activity. However, by the enhanced mRNA and protein content of Ki67 at the same time, we supposed a compensatory elevation in cell proliferation stimuli. From our perspective, persists unexplained the discrepancy in proliferative cell markers found in NP kidneys, being necessary for additional studies to elucidate each protein’s role.

Although inhibition of miR-199a-5p has been related to a reduction in cell proliferation and enhanced apoptosis (Sun et al., 2015), the expression curves showed an uncorrelated miR expression, cell cycle, and apoptosis-related proteins. The miR-199a-5p encoding and its predicted target, IGF1 mRNA, showed a similar modulation with upregulated expression of IGF1 mRNA in the different age times studied.

In the present work, among miRs, the miR-199a-5p expression was the most significantly related to mTOR mRNA encoding and protein immunoreactivity. The mTOR mRNA expression has a pronounced reduction in the time course from 17GD to 7DL LP offspring kidney. The upstream of mTOR mRNA signaling and the IGF1 were upregulated at the LP compared to NP beyond 17GD of age, but mTOR mRNA expression was unchanged. Conversely, in the 7DL LP kidney, the mTOR mRNA was significantly reduced compared to age-matched NP progeny. On the other hand, mTOR immunoreactivity in 21GD and 7DL kidneys was notably enhanced compared to NP offspring. Thus, we can suppose that a post-translational factor can be maintaining the raised protein level.

The miR-298-5p and its predicted targets showed a positive regulation of Bcx and negative of IGF1 mRNA. In this way, the miR-298-5p acted to enhance IGF1 and, consequently, mTOR signaling. mTOR signaling plays a central role in sensing response to intracellular nutrient availability (Marshall, 2006; Nijland et al., 2007). The kidneys transcriptome from a fetal baboon, whose mothers are submitted to regular content or nutrient-restricted (70% reduced fed), demonstrated that the mTOR signaling pathway is central to induce a reduction in the nephron number in this model (Nijland et al., 2007). Although it is widely known that mTORC1 has an essential role in embryo development, keep completely unclear the complex mechanisms in stress conditions (Gürke et al., 2016).

Nakagawa et al., 2015 have shown that miR 199a-5p is related to activation of the WNT pathway regulating vascular and nephron development. Here, we can suppose that miR-199a-5p positively modulated VEGF expression (mRNA and protein) in the LP kidneys. VEGF signaling is a downstream event of the mTOR pathway, but, in the present study, the VEGF mRNA was not accompanied by the mTOR mRNA encoding; however, it occurs in parallel to VEGF immunoreaction. Kitamoto et al. (1997) studied *in vivo* the role of VEGF in kidney development by blocking the endogenous VEGF activity in newborn mice. They showed a reduced nephron number and abnormal glomeruli. The increased VEGF reactivity observed in 21GD, and 7DL kidneys from the LP progeny indicate a possible compensation of peritubular and glomerular capillary development; once in the 17GD LP kidneys, the low expression could impair vascular development.

The present study of miR let7a-5p and its predicted mRNA targets observed a reciprocal behavior of downregulated c-myc and Six2 upbeat mRNA encoding in 21GD LP kidneys fetus. Besides the 32% increase, Six2 was expressing cells in the CM of 21GD LP kidneys, a 70% significantly enhanced CM area cannot be explained only by cell number. Beyond the interphase nuclei characteristics, the cells at LP CM from 21GD kidneys are more prominent than those observed in the NP progeny. When in the G1 phase, the cells increase protein synthesis for DNA replication (S phase) and growth, and then, in the G2 phase, cells grow before mitosis. After mitogenic stimuli of cells in G1, cyclin D bind to cyclin-dependent kinases (CKD) and activate a downstream process that led to the S phase, which is regulated by the cyclin E-CDK2 complex, while the G2/M transition is under control of cyclin B-CDK1 complex (Mens and Ghanbari, 2018). Thus, in 21GD LP kidneys, we can modulate other cyclins (D, E, or E), leading to a possible cell cycle arrest. Another intriguing observation in the 21GD LP kidneys is the strong reactivity of Six2, a marker of undifferentiated state, in cells of vesicles. Our results demonstrate a progenitor cell proliferation and self-renewal pathways activation, in the whole-kidney tissue, with cells supposedly in the interphases. These LP fetus cells are significant increased than observed in NP progeny following upregulated growth and protein synthesis. The upregulation of the cell differentiation and consumption was verified in 21GD LP kidneys. This result is incomprehensive, considering the increased Six2 positive cells in the CM and non-differentiated cells migrating to vesicles. Why and how a CM progenitor cell migrates to differentiating nephron structures remains unanswered.

We may suppose that miRNAs regulate stem cells’ mitotic cycle, and the let-7 miRNAs suppress their self-renewal (J Melton et al., 2010; Shim and Nam, 2016). The Lin28-Let-7 axis forms a crucial feedback loop in stem cell development (Zhou et al., 2015). The 21GD LP fetus kidneys presented upregulated miR let7a-5p and reduced c-myc mRNA expression and protein reactivity, which is permissive to increased Six2 expression observed. We could suggest that both proliferation and differentiation of stem cells may have been blocked. Reinforcing this hypothesis, the miR 127-3p and let7a-5p in 21GD LP fetus kidneys up-regulated PRDM1 mRNA, a predicted target of the miRNAs. Prdms promote and maintain stem and primordial germ cell identity (To review, Hohenauer and Moore, 2012). Prdm can repress TGFβ downstream signaling (Alliston et al., 2005), and Prdm1 directly suppresses the proliferation factor *Myc* (Yu et al., 2000). Bcl6 also acts as a transcriptional repressor by a negative-feedback loop with Prdm1 (reviewed by Crotty et al., 2010). These components can also be performed to reduce stem cell differentiation and proliferation in the nephrogenic zone of 21GD LP kidneys.

In the 7DL offspring from both experimental groups, higher altered miRNA expression was found due to raised nutrient content that led to the activation of pathways related to differentiation and progenitor cells’ consumption. Studies concerning the developmental origins of health and disease (DOHaD) have shown that the incompatibility between the environmental insults in the intrauterine phase and those conditions found after birth may result in health problems in adulthood (Gluckman et al., 2005; Gluckman and Hanson, 2006). In the present model, after delivery, the rat’s dams are fed with chow with standard protein supply, which leads to offspring catch-up growth extremely harmful to health in adult age. By the way, we hypothesize that a high number of miRNAs altered at 7DL LP kidneys concerning NP offspring are related to that accelerated growth. Thus, postnatal unrestricted food intake may induce nephron differentiation fastly and kidney vasculogenesis followed by mRNA and proteins overexpressed in 7DL LP compared to NP progeny.

*In conclusion*, the gestational protein-restricted intake induced differential kidney miRNA expression in the fetal and breastfeeding period. Considering these results seem to have a modulatory function on expressing specific genes and proteins associated with impaired nephrogenesis observed in this model.

We found clues to future in-depth analysis. It is essential to consider a complex and intricate gene expression network that miRNAs can regulate during development, integrating regulation and handling a large part of the transcriptome (Zhao and Srivastava, 2008). The cell cycle control and migration of CM undifferentiated cells need more investigations. VEGF protein expression and signaling also require focused future analysis. Whole-tissue homogenates do not reflect what can be found within the developing kidney’s different compartments. They are necessary studies in isolated cells from CM and BU for specifying where modulations are occurring.

## Data Availability Statement

The miRNA sequencing data has been deposited into the Sequence Read Archive repository (accession: PRJNA694197).

## Ethics Statement

The animal study was reviewed and approved by the Institutional Ethics Committee (CEUA/UNESP, Protocol #446). The general guidelines established by the Brazilian College of Animal Experimentation were followed throughout the investigation.

## Author Contributions

LB contributed to the data curation, investigation, formal analysis, methodology, visualization, and writing the original draft. GL contributed to the methodology and visualization. WS contributed to the methodology and supervision. AS contributed to the methodology and supervision. JG contributed to the formal analysis, methodology, visualization, and writing, review, and editing the manuscript. PB contributed to the conceptualization, formal analysis, funding acquisition, methodology, resources, supervision, visualization, and writing the original draft, and review and editing the manuscript. All authors contributed to the article and approved the submitted version.

## Conflict of Interest

The authors declare that the research was conducted in the absence of any commercial or financial relationships that could be construed as a potential conflict of interest.

## Abbreviations

Bax: Apoptosis regulator
Bcl2: B-cell lymphoma 2
Bcl6: B-cell lymphoma 6
Bim: or Bcl-2-like protein 11
BSA: Bovine serum albumin
cDNA: complementary deoxyribonucleic acid
CEUA/UNESP: Institutional Ethics Committee
c-Myc: regulator genes and MYC proto-oncogenes
CM: metanephros cap
c-ret: rearranged during transfection
DAB - 3,3′: diaminobenzidine tetrahydrochloride
DNA: deoxyribonucleic acid
GD: gestational days
GAPDH: Glyceraldehyde 3-phosphate dehydrogenase
GDNF: Glial cell line-derived neurotrophic factor
IGF1: Insulin-like growth factor 1
Ki67: a nuclear protein associated with cellular proliferation
let-7: lethal-7 (let-7) gene
Lin28b, Lin-28 Homolog B: suppressor of microRNA (miRNA) biogenesis
LP: gestational low-protein intake
Map2k2: mitogen-activated protein kinase kinase 2
*miRNA (miR)*: a small non-coding RNA molecule
*miRNA-Seq*: *miRNA sequencing*
mRNA: messenger ribonucleic acid
mTOR: mammalian target of rapamycin
NGS: Next Generation Sequencing
NOTCH1: single-pass transmembrane receptor protein
NP: normal protein intake
PCNA: Proliferating cell nuclear antigen
PRDM1: PR domain zinc finger protein 1
RIN: RNA Integrity Number
RT-qPCR: reverse transcription-polymerase chain reaction quantitative real-time
Six2: SIX homeobox 2
TGF β -1: transforming growth factor-beta 1
UB: ureter bud
U6 and U87: internal reference gene
Zeb1 and Zeb2: Zinc finger E-box -binding homeobox 1 and 2
21GD: 21th gestational day
7DL: 7 days of life kidney.

## References

[B1] Alliston T. Ko T. C. Cao Y. Liang Y. Y. Feng X. H. Chang C. (2005). Repression of bone morphogenetic protein and activin inducible transcription by Evi-1. *J. Biol. Chem.* 280 24227–24238. 10.1074/jbc.m414305200 15849193

[B2] Alvarez R. Jr. Wang B. J. Quijada P. J. Avitabile D. Ho T. (2019). Cardiomyocyte cell cycle dynamics and proliferation revealed through cardiac-specific transgenesis of fluorescent ubiquitinated cell cycle indicator (FUCCI). *J. Mol. Cell Cardiol.* 127 154–164. 10.1016/j.yjmcc.2018.12.007 30571978 PMC6588545

[B3] Ambros V. (2004). The functions of animal microRNAs. *Nature* 431 350–355. 10.1038/nature02871 15372042

[B4] Bartel D. P. (2004). MicroRNAs: genomics, biogenesis, mechanism, and function. *Cell* 116 281–297. 10.1016/s0092-8674(04)00045-5 14744438

[B5] Brown D. L. Walling B. E. Mattix M. E. (2016). “Urinary system,” in *Atlas of Histology of the Juvenile Rat*, eds Parker G. A. Picut C. A. (San Diego, CA: Elsevier).

[B6] Bushati N. Cohen S. M. (2007). microRNA functions. *Annu. Rev. Cell Dev. Biol.* 23 175–205. 10.1146/annurev.cellbio.23.090506.123406 17506695

[B7] Bustin S. A. Benes V. Garson J. A. Hellemans J. Huggett J. Kubista M. (2009). The MIQE guidelines: minimum information for publication of quantitative real-time PCR experiments. *Clin. Chem.* 55 611–622. 10.1373/clinchem.2008.112797 19246619

[B8] Chang T. C. Mendell J. T. (2007). microRNAs in vertebrate physiology and human disease. *Annu. Rev. Genomics Hum. Genet.* 8 215–239. 10.1146/annurev.genom.8.080706.092351 17506656

[B9] Chen G. Zhu W. Shi D. Lv L. Zhang C. Liu P. (2010). MicroRNA-181a sensitizes human malignant glioma U87MG cells to radiation by targeting Bcl-2. *Oncol. Rep.* 23 997–1003. 10.3892/or_00000725 20204284

[B10] Chu C. Rana T. M. (2007). Small RNAs: regulators and guardians of the genome. *J. Cell. Physiol.* 213 412–419. 10.1002/jcp.21230 17674365

[B11] Chu J. Y. S. Sims-Lucas S. Bushnell D. S. Bodnar A. J. Kreidberg J. A. Ho J. (2014). Dicer function is required in the metanephric mesenchyme for early kidney development. *AJP Renal Physiol.* 306 F764–F772. 10.1152/ajprenal.00426.2013 PMC3962603 24500693

[B12] Crotty S. Johnston R. J. Schoenberger S. P. (2010). Effectors and memories: Bcl-6 and Blimp-1 in T and B lymphocyte differentiation. *Nat. Immunol.* 11 114–120. 10.1038/ni.1837 20084069 PMC2864556

[B13] Fuentes-Baile M. Ventero M. P. Encinar J. A. García-Morales P. Poveda-Deltell M. Pérez-Valenciano E. (2020). Differential effects of IGF-1R small molecule tyrosine kinase inhibitors BMS-754807 and OSI-906 on human cancer cell lines. *Cancers (Basel)* 12:3717. 10.3390/cancers12123717 33322337 PMC7763458

[B14] Gluckman P. D. Hanson M. A. (2006). The consequences of being born small–an adaptive perspective. *Horm. Res.* 65 (Suppl. 3) 5–14. 10.1159/000091500 16612108

[B15] Gluckman P. D. Hanson M. A. Spencer H. G. (2005). Predictive adaptive responses and human evolution. *Trends Ecol. Evol.* 20 527–533. 10.1016/j.tree.2005.08.001 16701430

[B16] Grobstein C. (1955). Inductive interaction in the development of the mouse metanephros. *J. Exp. Zool.* 130 319–339. 10.1002/jez.1401300207

[B17] Gürke J. Schindler M. Pendzialek S. M. Thieme R. Grybel K. J. Heller R. (2016). Maternal diabetes promotes mTORC1 downstream signaling in rabbit preimplantation embryos. *Soc. Reprod. Fertil.* 151 465–476. 10.1530/REP-15-0523 26836250

[B18] Harvey S. J. Jarad G. Cunningham J. Goldberg S. Schermer B. Harfe B. D. (2008). Podocyte-specific deletion of dicer alters cytoskeletal dynamics and causes glomerular disease. *J. Am. Soc. Nephrol.* 19 2150–2158. 10.1681/ASN.2008020233 18776121 PMC2573015

[B19] Ho J. Pandey P. Schatton T. Sims-Lucas S. Khalid M. Frank M. H. (2011). The pro-apoptotic protein Bim is a microRNA target in kidney progenitor cells. *J. Am. Soc. Neph.* 22 1053–1063. 10.1681/ASN.2010080841 PMC3103725 21546576

[B20] Hohenauer T. Moore A. W. (2012). The Prdm family: expanding roles in stem cells and development. *Development* 139 2267–2282. 10.1242/dev.070110 22669819

[B21] Huang B. Liu Z. Vonk A. Zeng Z. Li Z. (2020). Epigenetic regulation of kidney progenitor cells. *Stem Cells Transl. Med.* 9 655–660. 10.1002/sctm.19-0289) 32163228 PMC7214665

[B22] Kim V. Han J. Siomi M. (2009). Biogenesis of small RNAs in animals. *Nat. Rev. Mol. Cell Biol.* 10 126–139. 10.1038/nrm2632 19165215

[B23] Kitamoto Y. Tokunaga H. Tomita K. (1997). Vascular endothelial growth factor is an essential molecule for mouse kidney development: glomerulogenesis and nephrogenesis. *J. Clin. Invest.* 99 2351–2357. 10.1172/jci119416 9153276 PMC508073

[B24] Langley-Evans S. C. (2006). Developmental programming of health and disease. *Proc. Nutr. Soc.* 65 97–105. 10.1079/pns2005478 16441949 PMC1885472

[B25] Li J. Y. Yong T. Y. Michael M. Z. Gleadle J. M. (2010). The role of microRNAs in kidney disease. *Nephrology (Carlton)* 15 599–608. 10.1111/j.1440-1797.2010.01363.x 20883280

[B26] Li W. Qiu X. Jiang H. Han Y. Wei D. Liu J. (2016). ScienceDirect downregulation of miR-181a protects mice from LPS-induced acute lung injury by targeting Bcl-2. *Biomed. Pharmacother.* 84 1375–1382. 10.1016/j.biopha.2016.10.065 27802900

[B27] Lucas A. (1998). Symposium: the effects of childhood diet on adult health and disease psychological influences on childhood diet 1. *J. Nutr.* 128 401S–406S. 10.1093/jn/128.2.401S 9478037

[B28] Lv X. Mao Z. Lyu Z. Zhang P. Zhan A. Wang J. (2014). miR181c promotes apoptosis and suppresses the proliferation of metanephric mesenchyme cells by targeting Six2 in vitro. *Cell Biochem. Funct.* 32 571–579. 10.1002/cbf.3052 25187057

[B29] Mackenzie H. S. Lawler E. V. Brenner B. M. (1996). Congenital oligonephropathy: the fetal flaw in essential hypertension? *Kidney Int. Suppl.* 55 S30–S34. 8743507

[B30] Marrone A. K. Ho J. (2014). MicroRNAs: potential regulators of renal development genes that contribute to CAKUT. *Pediatr. Nephrol.* 29 565–574. 10.1007/s00467-013-2599-0 23996519 PMC3944105

[B31] Maragkakis M. Alexiou P. Grosse I. Hatzigeorgiou A. G. (2012). Functional microRNA targets in protein coding sequences. *Bioinformatics* 28 771–776. 10.1093/bioinformatics/bts043 22285563

[B32] Marshall S. (2006). Role of insulin, adipocyte hormones, and nutrient-sensing pathways in regulating fuel metabolism and energy homeostasis: a nutritional perspective of diabetes, obesity, and cancer. *Sci. STKE* 2006:re7. 10.1126/stke.3462006re7 16885148

[B33] Matsubara J. Takashima A. Kato K. Hamaguchi T. Shirao K. Shimada Y. (2009). Relationships of insulin-like growth factor-1 receptor and epidermal growth factor receptor expression to clinical outcomes in patients with colorectal cancer. *Oncology* 76 42–48. 10.1159/000178164 19033715

[B34] Melton C. Judson R. L. Blelloch R. (2010). Opposing microRNA families regulate self-renewal in mouse embryonic stem cells. *Nature* 463 621–626. 10.1038/nature08725 20054295 PMC2894702

[B35] Mens M. M. J. Ghanbari M. (2018). Cell cycle regulation of stem cells by microRNAs. *Stem Cell Rev. Rep.* 14 309–322. 10.1007/s12015-018-9808-y 29541978 PMC5960494

[B36] Mesquita F. F. Gontijo J. A. Boer P. A. (2010a). Expression of renin-angiotensin system signalling compounds in maternal protein-restricted rats: effect on renal sodium excretion and blood pressure. *Nephrol. Dial. Transplant.* 25 380–388. 10.1093/ndt/gfp505 19793932

[B37] Mesquita F. F. Gontijo J. A. R. Boer P. A. (2010b). Maternal undernutrition and the offspring kidney: from fetal to adult life. *Braz. J. Med. Biol. Res.* 43 1010–1018. 10.1590/s0100-879x2010007500113 21049242

[B38] Moore M. W. Klein R. D. Farinas I. Sauer H. Armanini M. Phillips H. (1996). Renal and neuronal abnormalities in mice lacking GDNF. *Nature* 382 76–79. 10.1038/382076a0 8657308

[B39] Monk C. Spicer J. Champagne F. A. (2012). Linking prenatal maternal adversity to developmental outcomes in infants: the role of epigenetic pathways. *Dev. Psychopathol.* 24 1361–1376. 10.1017/S0954579412000764 23062303 PMC3730125

[B40] Nagalakshmi V. K. Ren Q. Pugh M. M. Valerius M. T. McMahon A. P. Yu J. (2011). Dicer regulates the development of nephrogenic and ureteric compartments in the mammalian kidney. *Kidney Int.* 79 317–330. 10.1038/ki.2010.385 20944551 PMC3214622

[B41] Nakagawa N. Xin C. Roach A. M. Naiman N. Shankland S. J. Ligresti G. (2015). Dicer1 activity in the stromal compartment regulates nephron differentiation and vascular patterning during mammalian kidney organogenesis. *Kidney Int.* 87 1125–1140. 10.1038/ki.2014.406 25651362 PMC4449790

[B42] Nijland M. J. Schlabritz-loutsevitch N. E. Hubbard G. B. Nathanielsz P. W. Cox L. A. (2007). Non-human primate fetal kidney transcriptome analysis indicates the mammalian target of rapamycin (mTOR) is a central nutrient-responsive pathway. *J. Physiol.* 579 643–656. 10.1113/jphysiol.2006.122101 17185341 PMC2151384

[B43] Nilsen T. W. (2007). Mechanisms of microRNA-mediated gene regulation in animal cells. *Trends Genet.* 23 243–249. 10.1016/j.tig.2007.02.011 17368621

[B44] Pan C. Chen H. Wang L. Yang S. Fu H. Zheng Y. (2012). Down-regulation of MiR-127 facilitates hepatocyte proliferation during rat liver regeneration. *PLoS One* 7:e39151. 10.1371/journal.pone.0039151 22720056 PMC3376093

[B45] Pfaffl M. W. (2001). A new mathematical model for relative quantification in real-time RT–PCR. *Nucleic Acids Res.* 29:e45. 10.1093/nar/29.9.e45 11328886 PMC55695

[B46] Pan X. Karner C. M. Carroll T. J. (2017). Myc cooperates with beta-catenin to drive gene expression in the nephron progenitor cells. *Development* 144 4173–4182. 10.1242/dev.153700 28993399 PMC5719246

[B47] Phua Y. L. Chu J. Y. S. Marrone A. K. Bodnar A. J. Sims-Lucas S. Ho J. (2015). Renal stromal miRNAs are required for normal nephrogenesis and glomerular mesangial survival. *Physiol. Rep.* 3:e12537. 10.14814/phy2.12537 26438731 PMC4632944

[B48] Pichel J. G. Shen L. Sheng H. Z. Granholm A. C. Drago J. Grinberg A. (1996). Defects in enteric innervation and kidney development in mice lacking GDNF. *Nature* 382 73–76. 10.1038/382073a0 8657307

[B69] Reczko M. Maragkakis M. Alexiou P. Grosse I. Hatzigeorgiou A. G. (2012). Functional microRNA targets in protein coding sequences. *Bioinformatics* 28, 771–776. 10.1093/bioinformatics/bts043 22285563

[B49] Sanchez M. P. Silos-Santiago I. Frisen J. He B. Lira S. A. Barbacid M. (1996). Renal agenesis and the absence of enteric neurons in mice lacking GDNF. *Nature* 382 70–73. 10.1038/382070a0 8657306

[B50] Saxen L. Sariola H. (1987). Early organogenesis of the kidney. *Pediatr. Nephrol.* 1 385–392. 10.1007/BF00849241 3153305

[B51] Schreuder M. Delemarre-Van De Waal H. Van Wijk A. (2006). Consequences of intrauterine growth restriction for the kidney. *Kidney Blood Press. Res.* 29 108–125. 10.1159/000094538 16837795

[B52] Schuchardt A. D’Agati V. Larsson-Blomberg L. Costantini F. Pachnis V. (1994). Defects in the kidney and enteric nervous system of mice lacking the tyrosine kinase receptor Ret. *Nature* 367 380. 10.1038/367380a0 8114940

[B53] Scorrano L. Korsmeyer S. J. (2003). Mechanisms of cytochrome c release by proapoptotic BCL-2 family members. *Biochem. Biophys. Res. Commun.* 304 437–444. 10.1016/s0006-291x(03)00615-6 12729577

[B54] Sene L. B. Mesquita F. F. de Moraes L. N. Santos D. C. Carvalho R. Gontijo J. A. (2013). Involvement of renal corpuscle microRNA expression on epithelial-to-mesenchymal transition in maternal low protein diet in adult programmed rats. *PLoS One* 19:e71310. 10.1371/journal.pone.0071310 23977013 PMC3747155

[B55] Sene L. B. Rizzi V. H. G. Gontijo J. A. R. Boer P. A. (2018). Gestational low-protein intake enhances whole-kidney miR-192 and miR-200 family expression and epithelial-to-mesenchymal transition in rat adult male offspring. *J. Exp. Biol.* 22:221. 10.1242/jeb.171694 29789348

[B56] Sene L. B. Scarano W. R. Zapparoli A. Gontijo J. A. R. Boer P. A. (2021). Impact of gestational low-protein intake on embryonic kidney microRNA expression and in nephron progenitor cells of the male fetus. *PLoS One* 16:e0246289. 10.1371/journal.pone.0246289 33544723 PMC7864410

[B57] Shim J. Nam J. W. (2016). The expression and functional roles of microRNAs in stem cell differentiation. *BMB Rep.* 49 3–10. 10.5483/bmbrep.2016.49.1.217 26497582 PMC4914210

[B58] Sun H. Zhou C. Fu L. (2015). Inhibition of MiR-199a-5p reduced cell proliferation in autosomal dominant polycystic kidney disease through targeting CDKN1C. *Med. Sci. Monit.* 21 195–200. 10.12659/MSM.892141 25588980 PMC4304454

[B59] Sun N. Zhang L. Zhang C. Yuan Y. (2020). miR-144-3p inhibits cell proliferation of colorectal cancer cells by targeting BCL6 via inhibition of Wnt/β-catenin signaling. *Cell Mol. Biol. Lett.* 17:19. 10.1186/s11658-020-00210-3 32206063 PMC7079415

[B60] Valsecchi M. E. McDonald M. Brody J. R. Hyslop T. Freydin B. Yeo C. J. (2012). Epidermal growth factor receptor and insulinlike growth factor 1 receptor expression predict poor survival in pancreatic ductal adenocarcinoma. *Cancer* 118 3484–3493. 10.1002/cncr.26661 22086503

[B61] Vasudevan S. Tong Y. Steitz J. A. (2007). Switching from repression to activation: microRNAs can up-regulate translation. *Science* 318 1931–1934. 10.1126/science.1149460 18048652

[B62] Vlachos I. S. Hatzigeorgiou A. G. (2013). Online resources for miRNA analysis. *Clin. Biochem.* 46 879–900. 10.1016/j.clinbiochem.2013.03.006 23518312

[B63] Wei J. Zhang Y. Luo Y. Wang Z. Bi S. Song D. (2014). Aldose reductase regulates miR-200a-3p/141-3p to coordinate Keap1-Nrf2, Tgf1/2, and Zeb1/2 signaling in renal mesangial cells and the renal cortex of diabetic mice. *Free Radic. Biol. Med.* 67 91–102. 10.1016/j.freeradbiomed.2013.10.811 24161443

[B64] Xiang C. Cui S. Ke Y. (2016). MiR-144 inhibits cell proliferation of renal cell carcinoma by targeting mTOR. *J. Huazhong Univ. Sci Technolog. Med. Sci.* 36 186–192. 10.1007/s11596-016-1564-0 27072960

[B65] Yu J. Angelin-Duclos C. Greenwood J. Liao J. Calame K. (2000). Transcriptional repression by blimp-1 (PRDI-BF1) involves recruitment of histone deacetylase. *Mol. Cell. Biol.* 20 2592–2603. 10.1128/mcb.20.7.2592-2603.2000 10713181 PMC85475

[B66] Zhang Y. Miaomiao F. Zhang X. Huang F. Wu K. Zhang J. (2014). Cellular microRNAs up-regulate transcription via interaction with promoter TATA-box motifs. *RNA* 20 1878–1889. 10.1261/rna.045633.114 25336585 PMC4238354

[B67] Zhao Y. Srivastava D. (2008). A developmental view of microRNA function. *Trends Biochem. Sci.* 32 189–197. 10.1016/j.tibs.2007.02.006 17350266

[B68] Zhou Y. Li Y. S. Bandi S. R. Tang L. Shinton S. A. Hayakawa K. (2015). Hard Lin28b promotes fetal B lymphopoiesis through the transcription factor Arid3a. *J. Exp. Med.* 212 569–580. 10.1084/jem.20141510 25753579 PMC4387290

